# Optimized cascaded regulation strategy for robust automatic generation control in renewable-integrated power networks

**DOI:** 10.1038/s41598-025-31703-w

**Published:** 2025-12-27

**Authors:** Kareem M. AboRas, Mohammed Hassan EL-Banna, Ahmed M. EL-Wakil, Muhammad R. Hammad

**Affiliations:** Department of Electrical Power and Machines, Faculty of Engineering, Alexandria University, Alexandria, 21544 Egypt

**Keywords:** AGC, LFC-AVR, Differentiated creative search algorithm, FOPI-TID^μ^-PIDA controller, Hybrid three-area system, Green energy sources, Energy science and technology, Engineering

## Abstract

Ensuring stability of both voltage and frequency in linked power networks (LPNs) is a critical challenge, primarily due to their nonlinear dynamics and load variability. Due to the high penetration of intermittent renewable energy sources, traditional Load Frequency Control (LFC) and Automatic Voltage Regulation (AVR) schemes often struggle to ensure fast, robust, and coordinated regulation in modern multi-area hybrid grids. To address these limitations, this research introduces a novel cascaded control architecture developed for Load Frequency Control (LFC) and Automatic Voltage Regulation (AVR) within a three-area hybrid LPN comprising thermal, wind, hydro, photovoltaic, and diesel generation sources. The proposed framework integrates three cascaded regulators: FOPI, TIDμ, and PIDA. Combining strengths of the three controllers provides better transient response, higher robustness against system uncertainties, and Improved steady-state accuracy. Also, for better performance, the parameters of the proposed controller are optimally selected using a recent developed optimization algorithm called Differential Creative Search (DCS). All simulations were carried out in MATLAB/Simulink environment. The obtained results are comprehensively compared to results obtained by utilizing other algorithms, Artificial Ecosystem-based Optimization (AEO), Dandelion Optimizer (DO), and the Runge–Kutta Optimization (RUN) algorithm. Results indicate that the DCS algorithm achieved the superior outcome, attaining the lowest objective function value of 0.0507, surpassing AEO, DO, and RUN with values of 0.0706, 0.0789, and 0.0649, respectively. Furthermore, the proposed controller was benchmarked against advanced control strategies such as FOPI–PI, TFOIDFF, and FOPI–PIDD2, yielding improvements in objective function values by 28.89%, 54.89%, and 26.42%, respectively. The simulation findings demonstrate that the FOPI–TIDμ–PIDA controller ensures significantly reduced overshoot by less than 0.12 Hz, faster settling times by less than 9.4 s, and enhanced voltage–frequency regulation even under ±25% variations in system parameters. Collectively, these results Proves the robustness, adaptability, and effectiveness of the proposed controller in advancing the stability and resilience of sustainable hybrid linked power networks.

## Introduction

### Background

During recent decades, the extensive reliance on fossil fuels has significantly raised carbon dioxide emissions, thereby intensifying the impacts of climate change. Moreover, electricity generation from conventional power plants is associated with high costs. These challenges have accelerated the adoption of renewable energy sources as a sustainable and environmentally friendly alternative. Modern power grids contain various techniques of generation power units interconnected to deliver highly quality power to meet the load variation demands. Generation units are generally grouped into coordinated control areas, where all generators within a specific area operate in synchronization. These regulated zones are networked through transmission links, enabling the Power transfer among them. Given that system load is continually fluctuating, ensuring grid steadiness, which relies on effectively regulating both frequency and terminal voltage, presents a considerable challenge. Frequency control is achieved by mitigating the imbalance between generated and demanded active power is managed by regulating the generator’s speed governor through load frequency control (LFC). The network’s terminal voltage is managed by the generator’s automatic voltage regulator (AVR), which modifies the excitation current in the generator field.

### Literature review

Concept of (LFC) in thermal power systems was explored first and later expanded to interconnected systems multi area that include different types of generation units^1^. Multi-region thermal plants were examined using single-stage turbine structures^2,3^, Generation Rate Constraints (GRC) was applied either by including or excluding the nonlinearity. Hydrothermal units equipped with reheat turbines were incorporated to three areas with equal generation capacities^4–6^, however, analysis didn`t consider the system nonlinearities. Including other renewable energy resources such as wind, Photovoltaic, fuel cells, and aqueous electrostatic reaction were considered as an expansion for analysis. Also, penetrating of gas and nuclear power generations besides the conventional hydro-thermal system can be considered^7,8^. Although the load frequency control is addressed, investigations ignored the crucial role of Automatic Voltage regulators (AVRs).

Integrated power system models were examined concentrating only on single-area systems^9,10^. On the other hand^11^, investigated systems containing three different areas overlooking system nonlinearity using traditional controller of I/PI. Integrated three-region power network with multiple Power output unit types is analyzed, considering the LPN. In loops of both LFC and AVR, it is crucial to manage deviations of frequency and terminal voltage under load demand fluctuations^12^. The study exploited the advanced degree of freedom (DOF) as a traditional controller. Another traditional controller like PID was utilized in^2,3,13^. Furthermore, cognition-driven approaches, including fuzzy PI and fuzzy PID controllers^6,14,15^, along with FAMCON toolbox-based strategies like fractional-order FOPI, TID and FOPID controllers^7,16^, have been implemented. The effectiveness of these regulators is largely influenced by the precise tuning of their gain values, achievable through soft computing techniques. Various optimization algorithms have been employed for this purpose, including Differentiated Creative Search (DCS)^16^, Particle Swarm Optimization (PSO)^17^, Wild Horse Optimizer (WHO)^18^, Dandelion Optimizer (DO)^19^, Triangulation Topology Aggregation Optimizer (TTAO)^20^, Runge-Kutta optimizer (RUN)^21^. While these optimization algorithms offer significant benefits, they often face limitations, including slow convergence, the probability of getting stuck into local minimal solution, and the balance between exploration and exploitation. The complexity is how to overcome these limitations considering that small changes in algorithm parameters can lead to large disturbances in dynamic behavior of the whole system. Therefore, a robust and self-sufficient optimization algorithm is essential to effectively face these challenges. Voltage and frequency stabilization units highlights the deployment of traditional robust control strategies which are enhanced either by DCS optimization techniques^22–24^ or fuzzy logic control^25,26^. Although conventional PID controller plays a great role as due to its robust design and low cost, it depends on trial and error when dealing with system nonlinearities and disturbances, hence different optimization techniques have been employed for optimal selection of key parameters of PID to address the automatic voltage regulator (AVR) problem^27,28^. Similarly, FOPID^29^ and Dual-degree state-driven PI controller^30^ controllers were utilized in the same manner. The evaluation shows the way taken to deal with the AVR challenge across different parameter variations^31^. Also, coupling between frequency and voltage were discussed for control system including different techniques such as FOPI-PI^32^, TFOIDFF^33^, and FOPI-PIDD^2^^34^. Considering the same problem was investigated considering sustainable energy supplies like wind^35,36^, Solar heat energy^37^, Terrestrial heat energy^38^. Moreover, penetration of energy storage devices such as RFB^39^ and other storage devices^40,41^ were explored. Furthermore, traditional controllers introduce limitations in handling system uncertainties and nonlinearities, such as the fluctuations^42^. Process performed by aid of resilience regulator reinforced with fraction calculus^43^, outlining of how to utilize advantages of each own regulator to boost upgrade traditional controller approaching to performances of advanced controller with retain advantages of smoothing management of traditional controllers. However, recent studies explored the use of sophisticated and more resilient control techniques for power grid stabilization challenges^44–46^. For instance, the critical role of frequency harmonization in power networks that rely on renewable energy sources were considered^47–49^.

Fractional-order and hybrid intelligent controllers for tackling complex control and optimization challenges were explored in various studies. a modified swarm-based intelligent controller for enhancing microgrid regulation was introduced in^50^, advanced fractional-order fuzzy PID controller was incorporated to hybrid learning strategies tailored for AGC systems^51^. A chaos–fuzzy hybrid fractional controller was developed to in interconnected power grids^52^. Robustness of hybrid bio-inspired optimization algorithms under complex system uncertainties was analyzed^53^. Deep neuro-fuzzy architectures is applied to deal to nonlinear and grid-connected energy systems^54^. For the aspect of smart grid applications, enhanced PSO is incorporated to chaotic mapping^55^. Advanced AGC strategies for hybrid power systems integrating variable renewable sources^56^. Neuro-optimization-based dynamic load frequency controller, emphasizing adaptability under variable operating conditions^57^. Real-time impacts of nonlinear control for voltage stability in power electronics-dominant environments was addressed^58^. The efficacy of bio-inspired metaheuristics for high-dimensional controller parameter tuning was demonstrated^59^. Surrogate-assisted optimization to streamline energy-efficient PID tuning was proposed^60^. A novel hybrid ANFIS-PSO stabilizer, reflecting a shift toward intelligent, adaptable, and hybridized grid control architectures was introduced in^61^. A modified chaos quasi-opposition-based sea horse optimization (CQOSHO) approach has been addressed for Automatic generation control (AGC) control^62^. a modified MPC controller has been proposed and evaluated and gives promising results^63^ by improving the system undershoot, overshoot, and settling time and enhancing performance by 45%. Also, Quasi Opposition based Whale Algorithm (QOWOA) is considered for AGC for two interconnected-area power system containing thermal unit and Solar generation, with utilization of ultra capacitor^64^. In^65^, three controllers of TID, ID-T, and PID-T are applied to a power plant contains various nonlinearities like generation rate constraint (GRC), governor dead band (GDB), and boiler dynamics. The system includes three energy sources, thermal, gas, and hydro. The result proved superiority of the proposed controller.

### Contribution of the paper

This study aims to enhance the stability of system voltage and frequency under renewable power perturbations. A novel control strategy is introduced through the development of the Fractional Order Proportional Integral-Tilt Integral Fractional Derivative-Proportional Integral Derivative Acceleration (FOPI-TID^μ^-PIDA) controller. The proposed FOPI-TID^μ^-PIDA controller has been meticulously calibrated using the Differentiated Creative Search (DCS) algorithm to ensure robust Frequency regulation and system robustness under varying of abnormal conditions. The key contributions of this work are summarized as follows:

- A novel coordinated control strategy is introduced by integrating a fractional-order FOPI controller, a TIDμ controller, and a PIDA controller, with all parameters optimally tuned using the Dynamic Contraction Search (DCS) algorithm. This hybrid control structure has not been previously applied to the joint design of LFC and AVR loops in multi-area power systems.
- The proposed controller aims to simultaneously enhance frequency stability and voltage regulation in a three-region integrated energy grid that includes conventional and renewable sources. This dual-loop optimization addresses limitations of existing approaches that treat LFC and AVR separately or rely on single-structure controllers.
- The proposed technique is evaluated under multiple practical scenarios, including large load disturbances and high fluctuations in renewable energy sources. Simulation results verify that the proposed method achieves faster settling time, lower overshoot, and higher robustness compared with conventional PID, FOPI, TID, and other controllers.

The paper is organized as follows: Following the presented introduction in Section I. Section II provides an overview for the architecture of system under study. Section III presents a detailed-explained methodology of the proposed DCS. Section IV discusses the configuration of the designed controller, including its setup and fine-tuning process. Section V presents the outcome findings, analysis, and relative discussions. Section VI provides a conclusion for the whole study, results, and comparisons, and addresses the areas of improvements and suggestions for future works.

### System modelling

As the global energy landscape shifts toward sustainable systems, maintaining voltage and frequency stability in hybrid multi-source power grids becomes increasingly critical and complex. Coordinated regulation of voltage and frequency in such systems particularly those with integrated renewable sources and storage faces challenges due to the interaction between the Load Frequency Control (LFC) and Automatic Voltage Regulator (AVR) loops^24^. In this context, a FOPI-TID^µ^-PIDA controller is proposed to simultaneously minimize voltage and frequency deviations in a composite grid combining conventional and renewable generation with storage units. The Area Control Deviation (ACD) and sensor outputs represent the measured outputs (OM) for the AVR and LFC loops, while the control signals to the governor and amplifier act as the controlled variables (CV). The dynamic behavior of these control loops is modeled using MATLAB Simulink, with detailed modeling steps discussed in the following sections.

### Joint AVR-LFC control scheme for hybrid interconnected power grids

Integrated control loops for voltage and frequency have been developed for a triple-power network area, each area comprises reheat, thermal and diesel units. as well as an independent renewable energy source. The model incorporates PV solar panel, Wind and hydropower generation units integrated into the combined AVR-LFC system. The capacity ratio between the areas is established at 1:2:4, with a load disturbance ($\Delta {P}_{L}$) of 0.1 pu implemented to Area 1. Subsection H details the System function modeling Of the AVR regulation cycle and its Interconnection alongside the loop of LFC^24^.

### LFC loop for thermal power plant

**Fig. 1:**
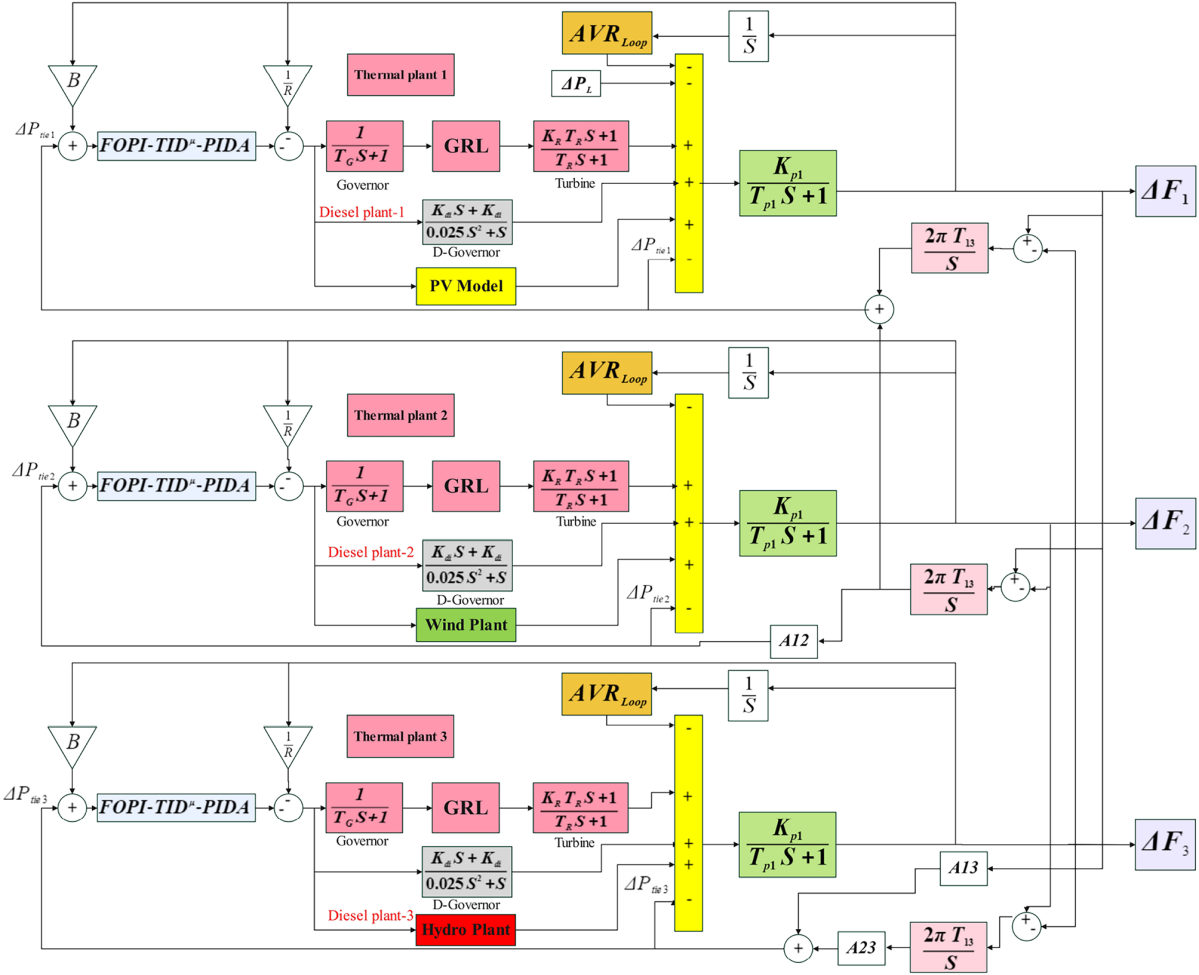
The studied triple-area green-based power system.

A thermal power generator based on a reheat turbine is modeled, with the turbine and governor configurations illustrated in Figure 1. 0.1 pu of step load disturbance (SLD) has been introduced as the Load fluctuation. The model has been built on linear based system function modeling for every element. Nonetheless, certain Intrinsic nonlinear characteristics, including Governor Dead Band (GDB), Generation Rate Limit (GRL), and time delays (TD), are consistently existing within the system and are analyzed in detail in Subsection G. The inclusion of the reheat turbine enhances overall efficiency by increasing the Average temperature. during the Heat input process. This improvement leads to a significant rise in entry of the temperature and expansion dynamics of the steam turbine effectiveness. The expansion takes place through a reheat cycle in two turbines, where the steam Stretches to a transitional pressure prior to boiler re-circulation. It is then repressurized at a steady pressure to its initial superheated temperature value. Reheating also resolves the problem regarding surplus moisture in the steam at the conclusion in the turbine expansion cycle. In Figure 1^8^, the measurements of time delay for the governor and turbine are shown, sequentially. The values of ${K}_{r}$ and ${T}_{r}$ represent the amplification factor and time delay for reheat turbine, the frequency variation in the region donated by $\Delta {f}_{x}$. The ensures for stable operation during abrupt load changes performed by governor droop control ($R$) within a thermal power Station by reducing reference speed as load grows.

The frequency offset coefficient (Of) Holds significant importance in determining the Area Control Deviation (ACD) for each area, which is used as the Input stimulus for traditional regulators and serves as the output measured (OM) For the Predictive Control Mode. The formula for ACD in ${k}^{th}$ area ($AC{D}_{x}$) is given in Equation (1)^8^.

1 $$ACD_{x} = \Delta P_{tie xy} + B \Delta f_{x}$$

The formula of deviations in tie-line power flow ($\Delta {P}_{tie kn}$) linked two selected regions ($k$ and $n$) appear in Equation (2).

2 $$\Delta {P}_{tie kn} =\frac{2\cdot \pi \cdot {P}_{tie xy}\left(\Delta {f}_{x}- \Delta {f}_{y}\right)}{ s}$$

Tie-line coupling factor (${P}_{tie xy}$) influenced by the metric of impedance (${X}_{xy}$) of the line transmission of linked area (and $y$). These equations and system architecture follow standard methodologies in power system frequency regulation studies^4,6,46^.

### LFC loop for photovoltaic (PV) plant representation

Solar PV power plant includes many photovoltaic panels, with each panel has several individual solar cells. So, an essential component of the solar power generation unit is the solar cell. The efficiency of a PV system’s energy fabrication is notably affected by weather conditions, making it behave unpredictably. As a result, substantial frequency variations caused Consequently, fluctuations in power output from solar PV systems can be assessed by considering variations caused by both uniform and non-uniform solar irradiance*.* Total current generated via photovoltaic cell is expressed in Equation (3)^5^. Meanwhile, the functional transfer equation representing the entire system, including the photovoltaic (PV) panel and electronic power components, provided in Equation (4)^3^.

3 $${I}_{pv}={I}_{L}-{I}_{0} \{exp [\frac{{V}_{pv}+{I}_{pv}{R}_{s}}{{n}_{cell}{V}_{th}}] - 1\} - \frac{{V}_{pv}+{I}_{pv}{R}_{s}}{{R}_{sh}}$$

Here, ${I}_{pv}$ is PV panel-generated current, ${V}_{pv}$ is $PV$ voltage, ${n}_{cell}$ Denotes the series-connected cell count, ${V}_{th}$ represents the thermal voltage, while the diode’s reverse leakage current is denoted as ${I}_{0}$.

4 $$T.F. of PV model = \frac{{a}_{pv}+s\cdot {b}_{pv}}{{s}^{2}+s\cdot {c}_{pv}+{d}_{pv}}$$

Here, ${a}_{pv}$, ${b}_{pv}$, ${c}_{pv}$, and ${d}_{pv}$ Represent fixed parameters used in modeling of the photovoltaic (PV) system obtained by curve fitting under step irradiance input^2,4^. This representation has been widely adopted in recent LFC simulations integrating solar PV^3,5^.

### LFC strategy for wind power plants

The model of a wind power generator for all regions have been developed using three fundamentals element., i.e., $Hydraulicpitchactuator$, $Pitch response$ Module, and the gain characteristics of the blades (GB)^25^. A simplified transfer function has been used to represent each component, as illustrated in Equations (5) and (6).

5 $$Hydraulicpitchactuator={K}_{w1}\frac{\left(1+s{T}_{w1}\right)}{\left(1+s{T}_{w2}\right)}$$

6 $$Pitch response = \frac{{K}_{w2}}{1 + s }$$

Here, ${K}_{w1}$ and ${K}_{w2}$ denote the gain values associated with the Pitch adjustment mechanism and the dynamics pitch model, respectively. ${T}_{w1}$ and ${T}_{w2}$ for the pitch actuator correspond to the time constant values. These models are consistent with recent literature on grid frequency support from wind farms^2,27^.

### Hydro plant based lfc system

The hydro energy facility is represented through a mechanical $Hydro governor$ and $Hydro turbine$^12^. The modeling of both components is defined in Equations (7) and (8).

7 $$Hydro governor = \frac{\left(1+{sT}_{R}\right)}{\left(1+s{T}_{RH}\right)\left(1+s{T}_{GH}\right)}$$

8 $$Hydro turbine = \frac{\left(1-s{T}_{w}\right)}{\left(1+0.5s{T}_{w}\right)}$$

${T}_{R}$, ${T}_{RH}$, and ${T}_{GH}$ represent the values of the time constants for $Hydro governor$ model. ${T}_{W}$ is the value of the time constants for $Hydro turbine$.

### Diesel plant based lfc system

The process of converting energy derived from fuel to mechanical energy subsequently into electrical energy is carried out by diesel generator sets. In this work, the diesel power plant Is depicted as a refined based transfer function second-degree model, being represented in Figure 1. The parameter $K{d}_{nn}$ denotes the diesel plant’s gain in the area ($k$).

### Nonlinear system modeling

Thermal power stations exhibit significant Nonlinear behaviors such as Governor Dead zone (GDZ) and Generation Rate limit (GRL), which are commonly observed in studies load frequency control. The GDZ means that the governor only responds to changes in speed after a certain threshold is exceeded, introducing a dead band where no immediate adjustments are made. The GRC limits how rapidly a turbine can alter its power output, imposing constraints on the rate of change. For this study, the GRC is set at 3% per unit MW per minute, and the GDB is set at 0.06%. Additionally, time delays in the Area control deviation (ACD) signal formed from frequency deviations and interconnected line power exchanges between neighboring areas can impact controller effectiveness and lead to increased system fluctuations. This study includes simulations that account for these time delays and nonlinearities to evaluate their effects on system performance.

### Evaluation of the avr loop and its relationship with the LFC loop

**Fig. 2:**
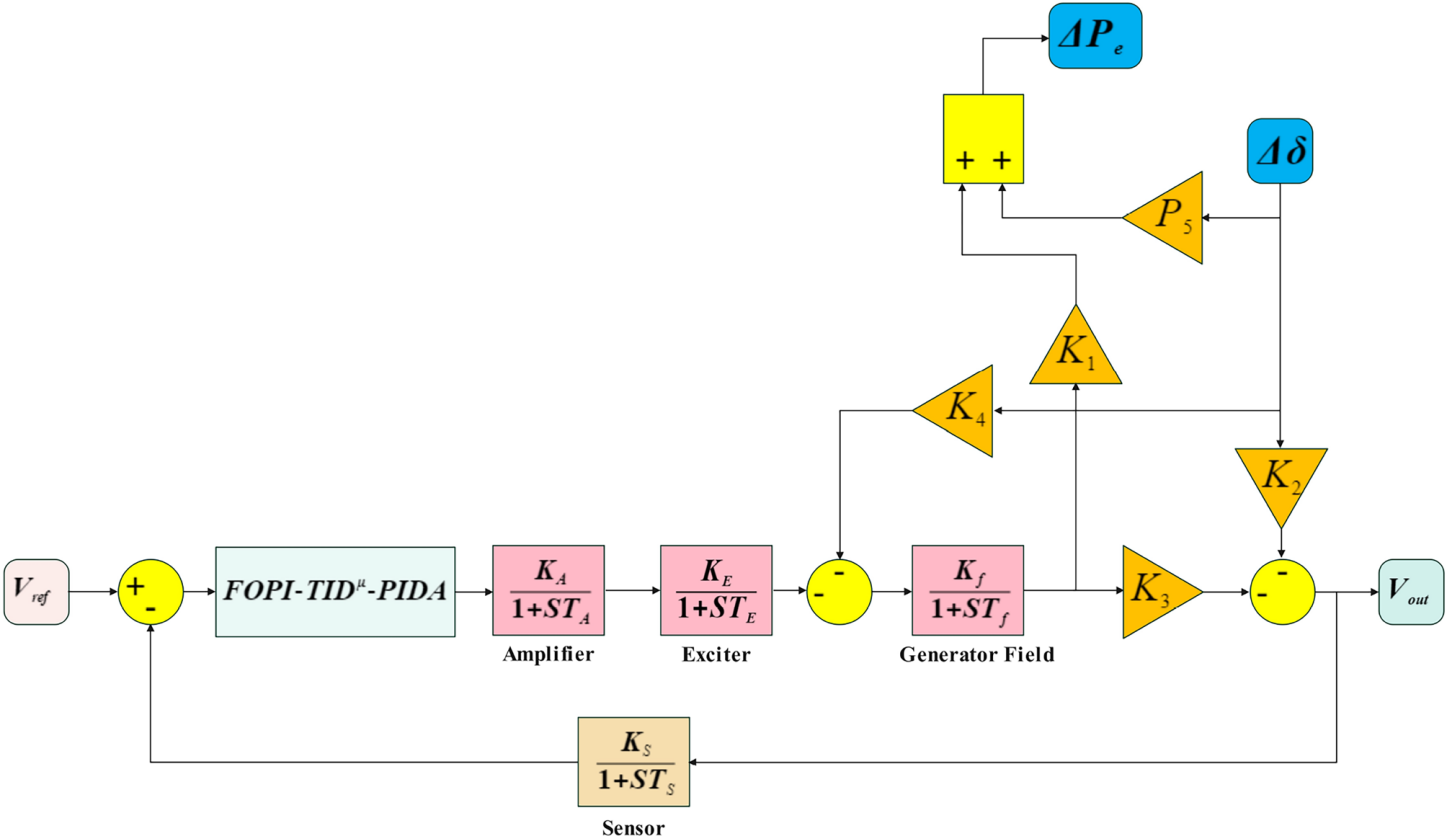
AVR loop’s block diagram representation with coupling coefficients.

Automatic Voltage Regulator (AVR) loop includes a signal booster, a stimulation device, a model of the field circuit, and sensor. all defined using first-order transfer functions with defined-time constants and gain values, as illustrated in Figure 2. Within this setup, the sensor output acts as the output measured (OM), and the amplifier receives an input of the control variable (CV). The components signal gain levels for AVR are higher compared to those in the Load Frequency Control (LFC) Feedback system, while the time constants for the LFC components are longer, making the LFC loop slower compared to the AVR loop. When real power demand changes, causing a drop in frequency system, the LFC loop attempts to counteract this by increasing generation, which in turn raises the terminal voltage. This rise in voltage results in a positive voltage error that the AVR loop addresses quickly due to its faster response time. Consequently, rapid response of the AVR loop is unlikely to be significantly influenced by the slower LFC loop. However, the LFC loop is set to be impacted via the AVR loop, as it changes in active power are directly related to terminal voltage, and even minor shifts in rotor angle can lead to substantial frequency changes Within the framework. The Gradual change in active power ($\Delta {P}_{e}$) depends on changes in the rotor angle ($\Delta \delta$) and the generated EMF (${E}_{1}$) as described in Equation (9)^12,24^.

9 $$\Delta {P}_{e}={P}_{s}\Delta \delta +{K}_{1}{E}_{1}$$

Equation (10) expresses the Deviation of output voltage (${\mathrm{V}}_{\mathrm{out}}$) concerning the Gradual rotor angle shift and induced electromotive force^13^.

10 $${V}_{out}={K}_{2}\Delta \delta +{K}_{3}{E}_{1}$$

Additionally, since the generated EMF (${E}_{1}$) is affected by changes in the Rotational angle of the rotor, it is essential to include its effect in the EMF equation given in Equation (11)^13^.

11 $${E}_{1} = \frac{{K}_{f}}{1 + {T}_{f} } [{V}_{field}-{K}_{4}\Delta \delta ]$$

In synchronous machines, the rotor angle deviation ($\Delta \delta$) and frequency deviation ($\Delta f$) are directly related through the time derivative. Frequency is the rate of change of rotor angle as described in Equation (12)^10^:

12 $$\Delta f\left(t\right)=\frac{1}{2\pi }\frac{d\left(\Delta \delta \left(t\right)\right)}{dt}$$

Taking Laplace Transform of both sides as in Equation (13)^10^:

13 $$\Delta F\left(s\right)=\frac{1}{2\pi }\bullet s\bullet \Delta \delta \left(s\right) \to \Delta \delta \left(s\right)=\frac{2\pi }{s}\bullet \Delta F\left(s\right)$$

In per-unit system (which is common in AGC models), the factor $2\pi$ is absorbed into system constants. Thus, for simplicity described in Equation (14):

14 $$\Delta \delta \left(s\right)=\frac{2\pi }{s}\bullet \Delta F\left(s\right)$$

It simply reflects the integral relationship between frequency and angle deviation.

${K}_{f}$ and ${T}_{f}$ are the gain and time constant values denotation of the power source’s field circuit, ${V}_{field}$ Serves as the excitation system’s output control system. $Ps$ Denotes the coefficient of synchronizing power and ${K}_{1}$, ${K}_{2}$, ${K}_{3}$ and ${K}_{4}$ Serve as the interconnection factors between the LFC and AVR loops. The Appendix contains all parameters used in AVR modeling. Block diagram for AVR loop is illustrated in Fig. 2^29^.

## The proposed control strategy and problem representation

### Differentiated creative search (DCS) optimizer

#### Inspiration

Analyzing several different Differential Evolution (DE) versions served as a source of motivation for DCS algorithm. In this part, the fundamental concepts that underpin the DE process are outlined. As the inquiry progresses, it becomes clear that DCS technique may not adhere to the principles of DE. Nevertheless, modern DE variations have increased the intricacy of the initial DE method, which was already rather complicated^16^. Although they are more powerful, these complicated variations pose difficulties in settings where memory resources are limited^22^.

#### Proposed concept

**Fig. 3:**
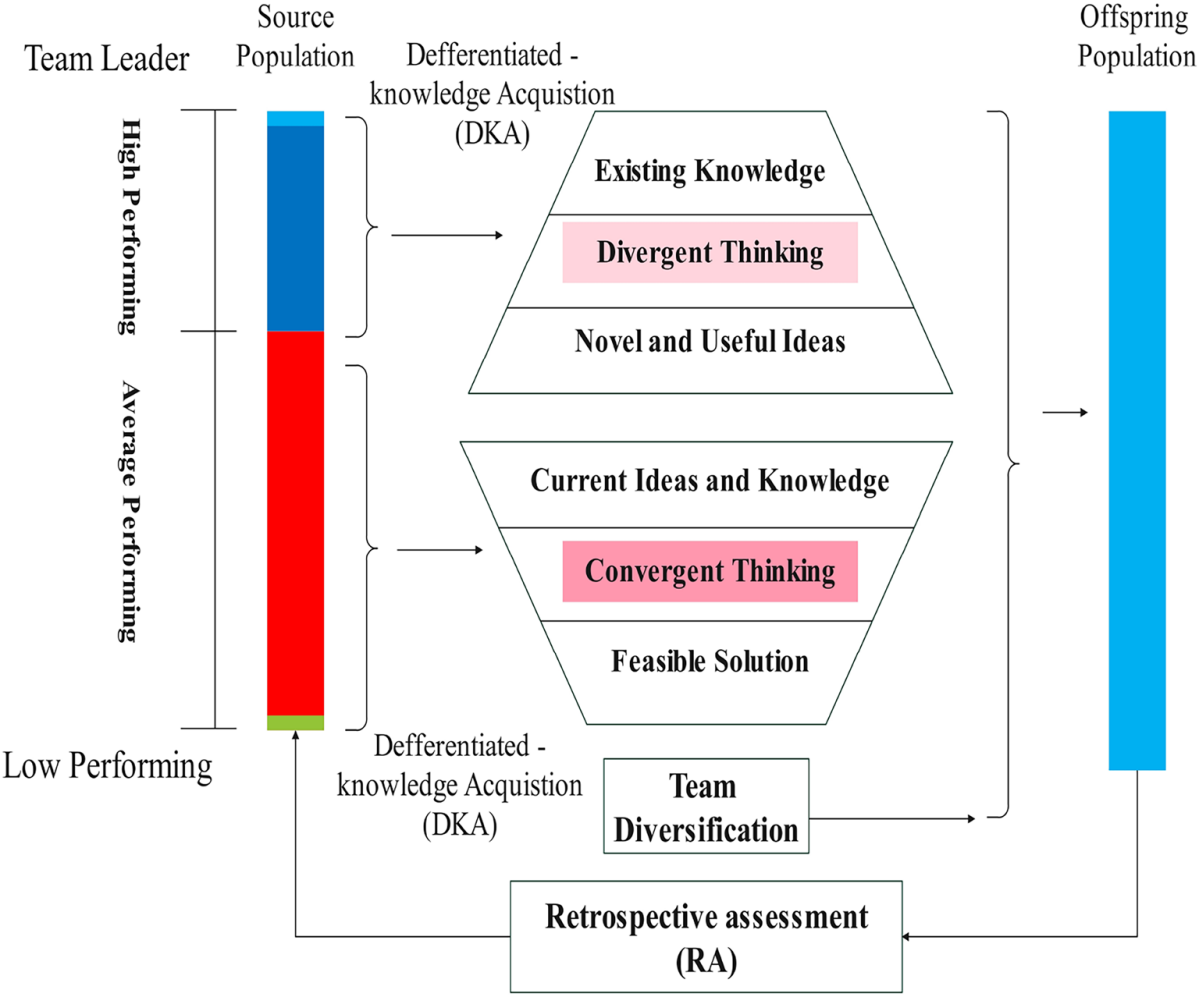
classifications of group participants according to DCS algorithm.

Differentiated Creative Search (DCS), illustrated in Figure 3, is an iterative model aimed at optimizing group effectiveness. It classifies group participants into three, assigning According to their performance tiers:

- **Top achievers:** These individuals are excellent at generating ideas through divergent thinking and independent learning, excelling in producing a wide range of Creative approaches.
- **Moderate achievers**: These members focus on refining ideas, by applying focused thinking to turn various concepts into feasible solutions.
- **Underperforming members:** Comparable to new recruits, as described in the WSO algorithm^23^, they contribute diversity, encourage probing, and attach new ideas to the team.

DCS operates through three main procedures:

- **Tailored Knowledge Acquisition (TKA):** This method categorizes Organize team members into clusters based on their skills, promoting development, and improving contributions.
- **Realistic Creativity:** This method encourages evolution via combining known information with creativity. It leverages Creative perspective of top performers and focused perspective of moderate achievers.
- **Reflective Evaluation (RE):** A monitoring process that tracks improvements and identifies top performers, providing critical data to inform future decisions and enhance team performance.

**Fig. 4:**
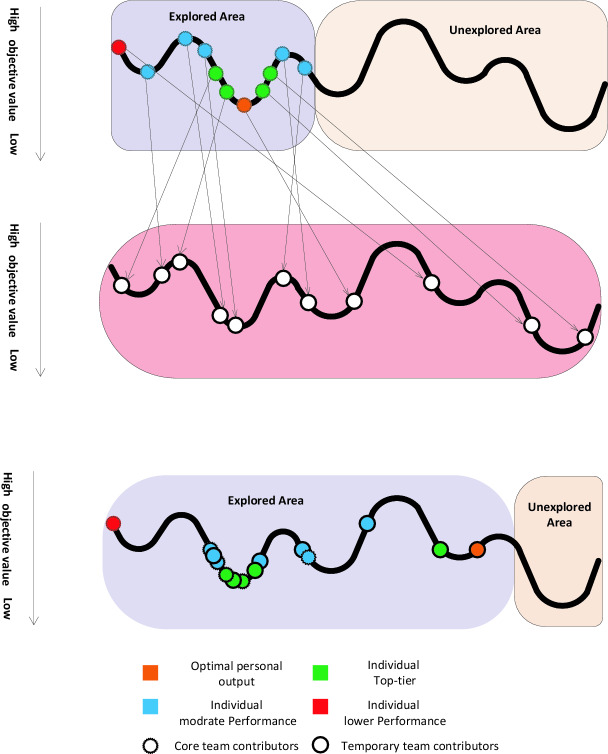
Visual image overview for the suggested strategies.

Figure 4 illustrates how divergent and convergent thinking actions play out. High performers are adept at generating varied Approaches and investigating various areas to avoid premature convergence on suboptimal solutions, while Moderate achievers specialize in improving ideas in promising areas. The next subsection provides further details on the DCS optimization.

#### Initial solution configuration

In the DCS algorithm, the adjustment process starts with an initial population of candidate solutions (referred to as team members) X, which are randomly generated within the bounds of the optimization problem, specifically Within the upper limit ($UB$) and lower limit ($LB$). The optimal approach identified During every iteration; it is regarded as the near-optimal solution. The set of team members $X$ is represented as follows:

15 $$X = \left[\begin{array}{cccc}{x}_{\mathrm{1,1}}& {x}_{1,d}& {x}_{1,D- 1}& {x}_{1,D}\\ {x}_{i,1}& {x}_{i,d}& {x}_{i,D- 1}& {x}_{i,D}\\ {x}_{NP- \mathrm{1,1}}& {x}_{NP- 1,d}& {x}_{NP- 1,D- 1}& {x}_{NP- 1,D}\\ {x}_{NP,1}& {x}_{NP,d}& {x}_{NP,D- 1}& {x}_{NP,D}\end{array}\right]$$

where ${X}_{i}=\left[xi1 xi,2 \cdots xi,D\right]$ In the matrix $X$ the $i$ ^th^ row corresponds to the $i$ ^th^ solution option, often referred to being the $i$ ^th^ entity. The element ${x}_{i,d}$ indicates the value at the $d$-th dimension of the $i$-th individual. Here, $NP$ denotes the total Set of proposed solutions, labeled as the set size, while $D$ quantifies the number of variables in the optimization problem. Each item in ${X}_{i}$ is initialized Using probabilistic selection Equation (16)^16^, as described below:

16 $${x}_{i,d}=L{B}_{d} + U (0, 1) \times (UB-L{B}_{d})$$

In this process, $U\left(\mathrm{0,1}\right)$ follows a uniform distribution. over the observed period $(0, 1)$, while $L{B}_{d}$ and $U{B}_{d}$ denote the minimum and maximum limits, respectively, for the $d$ -th dimension as specified by the optimization task. These equations and follow standard methodologies in DCS optimization studies^16,22^. classifications of group participants are shown in Figure 3^16^.

The suggested strategies can be visualized in Figure 4^16^. In part (a-upper), the dashed-dotted circles indicate group members during the ${t}^{th}$ iteration, with arrows indicating the approaches employed: Inventive ideation (green), Directed Ideation (blue), and Varied team assembly (red). In part (a-lower), Black points denote potential solutions explored in the search space after applying these approaches. In part (b), points represent the positions of individuals after Achievement appraisal, color labeled based on their target values: orange for the effective performers, green for high performers, blue for moderate performers, and red for low performers. Solid points indicate successful Temporary team contributors, while dotted-dashed circles represent successful Core team contributors.

Once initialized, all individual candidate solution ${x}_{i}$ is assessed to determine Goal or optimization metric. The solutions within $X$ are next sorted into increasing ordered depending on their objective values, with lower indices signifying better performance. At the onset of each generation, the ideal-performing solution ranks highest, while the worst performer ranks lowest. The standing of each $i$ th entity is determined by its performance, with a lower $i$ indicating a higher standing. The leading candidate is revised whenever a new, effective solution is identified.

#### Differentiated knowledge-acquisition (DKA)

The phases of diverse wisdom gathering are based on varying capacity for $k$ knowledge gathering and may be classified into three categories, contingent upon the person’s capability and performance stage. The initial category the high performer represents a top-tier individual., who acts as a generator of ideas and possesses a mindset conducive to producing a wide array of concepts; the second category is the moderate performer, who functions as a perspective refiner, scrutinising and contrasting various notion to identify the most effective solution; the third category is the low performer, who enhances The group’s heterogeneity and discovery via presenting novel notion and methodologies for Conflict resolution. The procedure for upgrading each individual’s position may be implemented using Equations (17) – (20)^16^, wherein the underperformers must acquire new information or experience to a greater extent than the high achievers.

17 $${\eta }_{i,t}= \frac{1}{2} \left(\left[U\left(\mathrm{0,1}\right)\times {\varphi }_{i,t}\right]+\left\{\begin{array}{c}1 if U(0, 1) \le {\varphi }_{i}\\ 0 otherwise\end{array}\right.\right)$$

18 $${\varphi }_{i,t} = 0.25 + 0.55 \times \sqrt{{R}_{i,t}/NP}$$

19 $${j}_{rand}\sim U (\{1, 2, ..., D\})$$

20 $${V}_{i,d }=\left\{\begin{array}{c}{v}_{i,d} ifU\left(\mathrm{0,1}\right)\le {\eta }_{i,t} or d={j}_{rand}\\ {x}_{i,d} otherwise\end{array}\right.$$

Here, ${V}_{i,d}$ denotes the $d$ th element (aspect) of the Temporary contributors ${v}_{i,t}$ and ${v}_{i, t}$ is the Temporary contributors ${v}_{i}$ at iteration $t$. ${x}_{i,d}$ refers to the $d$ th element (aspect) of ${X}_{i}$ in $X$. $U(0, 1)$ Indicates a steady spread throughout the span $(0, 1)$. ${\eta }_{i,t}$ represents the individual’s Discrete knowledge absorption rate (DKAR) at iteration $t$. ${j}_{rand}$ is an integer randomly chosen from $1$ to $D$, chosen once every $i$.

#### Realistic creativity

The diverse knowledge gathering categorises teams into poor, top, and moderate performers, with Innovative pragmatism derived from the lateral thinking of top performers and the direct thinking of ordinary workers. Innovative thinking and effective problem-solving are essential traits of great achievers. High performers not only provide a repository of knowledge, but each team member must continually acquire new information and expand their lateral thinking. For the typical performance, direct thinking depends on the expertise of the most proficient individual, supplemented by arbitrary inputs from two distinct members contributors. The positional update of an individual can be executed with the subsequent Equations (21)-(23)^16^:

21 $${Xnext }_{i,d}=\left\{\begin{array}{c}{X}_{r1,d}+L{K}_{i,d}(\alpha ,\sigma ), if i\le {n}_{gs}\\ {X}_{top ,d}+\gamma \cdot \left({X}_{r2,d}-{X}_{i,d}\right)+\omega \cdot \left({X}_{r1,d}-{X}_{i,d}\right), otherwise\end{array}\right.$$

22 $${n}_{gs}=max\left(6,\left[NP\cdot \frac{g}{3}\right]\right)$$

23 $$\gamma =0.1+0.518\cdot \left(1-{\left(\frac{t}{max \_it}\right)}^{0.5}\right)$$

${\mathrm{X}}_{\mathrm{r}1,\mathrm{d}}$​ represents the $\mathrm{d}$ th position of an arbitrarily selected individual from the set $\{\mathrm{1,2},\dots ,\mathrm{NP}\}$, while ${\mathrm{LK}}_{{{\mathrm{j}},{\mathrm{d}}}} \left( {{\upalpha },{\upsigma }} \right)$ is the Lévy stochastic number generator using control variables $\alpha$ and $\upsigma$. The term ${\mathrm{n}}_{\mathrm{gs}}$ refers to the number of top-performing individuals, determined by the Ideal demographic proportion, $\mathrm{g}$, which is an integer greater than or equal to six. ${\mathrm{X}}_{\mathrm{best},\mathrm{d}}$​ represents the $\mathrm{d}$ th position of the top-performing individual, and ${\mathrm{X}}_{\mathrm{r}2,\mathrm{d}}$ denotes the $\mathrm{d}$ th position of an individual randomly chosen from the set $\{{\mathrm{n}}_{\mathrm{gs}}+1,\dots ,\mathrm{NP}\}$. $\upomega$ Reflects the learning engagement state of an individual, typically set to a standard setting of 1. $\upgamma$ Signifies the individual’s factor at iteration $\mathrm{t}$, In which represents the iteration count, and $\mathrm{max}\_\mathrm{it}$ is the total iteration count. The $\upgamma$ coefficient quantifies the extent to which Group influences alter a person’s perspectives: boosted values imply a stronger dependence regarding participants contributors, while Reduced values signify increased autonomy. The DCS system swaps out underachieving individuals with new candidates to enhance the range of concepts created within the team. The equation for generating fresh additions is provided in formula (24)^16^*.*

24 $${X}_{i,d}=l{b}_{d}+{U}_{i}\left(\mathrm{0,1}\right)\cdot \left(u{b}_{d}-l{b}_{d}\right), if i=NP and {U}_{i}(\mathrm{0,1})<{p}_{c}$$

When ${p}_{c}$ is 0.5, the system reverts to selecting the remaining team participant. If ${U}_{i}(\mathrm{0,1})<{p}_{c}$​, it indicates Poor performance, and random setup is used to reinitialize the subject to baseline conditions

#### Edge management

The limit enforcement technique ensures that the formed concepts are both viable and aligned with practical constraints. If an individual’s position Along the $d$ ^th^ axis surpasses the bounds of feasibility, The system corrects the value by aligning it rounded to the closest boundary (Upper or lower) within the allowed limits within the dataset, as described in Equation (25)^16^. This approach is often identified a constraint management approach through interpolation., thereby eliminating the creation of impractical or unfeasible concepts.

25 $${v}_{i,d}= \left\{\begin{array}{c}\frac{{x}_{i,d}+L{B}_{d}}{2},{v}_{i,d}<L{B}_{d}\\ \frac{{x}_{i,d}+U{B}_{d}}{2},{v}_{i,d}>U{B}_{d}\end{array}\right.$$

${v}_{i,d}$ refers to the $d$-th element of trial member ${V}_{i,t}$ at iteration $t$. The Upper and lower limits for the $d$-th axis are $UB$ and, respectively.

#### Team diversification

To enhance team performance, the algorithm replaces low-performing members with new ones, as diverse teams are shown to be more effective Owing to their wider scope of ideas and Approaches to problem resolution. The formula used to create a new member is presented below:

26 $${V}_{NP}=LB + U\left(0, 1\right)\cdot (UB - LB)$$

Here, ${V}_{NP}$ is the $NP$-th temporary member in the set $V$, associated with ${X}_{NP}$. $U(0, 1)$ is a uniform distribution between 0 and 1, and $LB$ and $UB$ are the optimization problem’s bounds.

#### Retrospective assessment (RA)

Inspired by agile retrospectives, it helps teams by setting evaluation criteria and analyzing past performance to improve. It reveals strengths, weaknesses, and team dynamics, promoting continuous improvement. This study focuses on selection and tracking top performers within RA. The formula applied in the choosing procedure is provided below:

27 $${X}_{i,t+1}= \left\{\begin{array}{c}{V}_{i,t},f\left({V}_{i,t}\right)\le f\left({X}_{i,t}\right)\\ {X}_{i,t}, otherwise \end{array}\right.$$

where, ${X}_{i,t+1}$ denotes the $i$-th individual in $X$ at the $(t + 1)$ th iteration. ${V}_{i,t}$ represents the trial member of ${X}_{i,t}$, and ${X}_{i,t}$ represents the $i$-th individual at the $t$-th iteration. $f({V}_{i,t})$ and $f({X}_{i,t})$ are objective values of ${V}_{i,t}$ and ${X}_{i,t}$, In that order. The equation for evaluating and tracking the top performer is presented below:

28 $${X}_{top,t} = \left\{\begin{array}{c}{X}_{i,t+1},f\left({X}_{i,t+1}\right)<f\left({X}_{top,t}\right),\\ {X}_{top,t}, otherwise \end{array}\right.$$

where ${X}_{top, t}$ Signifies the top-performing individual in the $t$ ^th^ iteration, identified as ${X}_{i,t+1}$ Demonstrating the lowest value of the objective function ${X}_{i,t+1}$ represents the $i$-th individual in $X$ at the $\left(t+1\right)$-th iteration. $f\left({X}_{i,t+1}\right)$ and $f\left({X}_{top,t}\right)$ are objective values of ${X}_{i,t+1}$ and ${X}_{top, t}$, respectively. These equations and follow standard methodologies in DCS optimization studies^16,22,23^.

#### Computational complexity analysis

Understanding the computational burden of the proposed DCS algorithm is crucial for assessing its applicability to large-scale and real-time power systems. The time complexity of the DCS can be segmented into three core processes: initialization, generation of trial solutions, and population update. The total complexity of the algorithm is governed by the number of individuals in the population (NP), the dimensionality of the problem (D), and the number of iterations (T), leading to an overall time complexity of:

29 $$O(DCS) = O (NP \times D \times T)$$

This indicates that the required computational time increases linearly with these parameters. In terms of space complexity, the memory footprint is primarily influenced by the storage of the population matrix and trial solutions, yielding:

30 $$O\left(Space\right)= O\left(NP \times D\right)$$

This linear relationship ensures scalability, even when dealing with high-dimensional control problems in large-area power systems. All optimization computations are conducted offline, ensuring that the real-time operation of the controller remains computationally efficient.

#### Pseudocode of the DCS

**Fig. 5:**
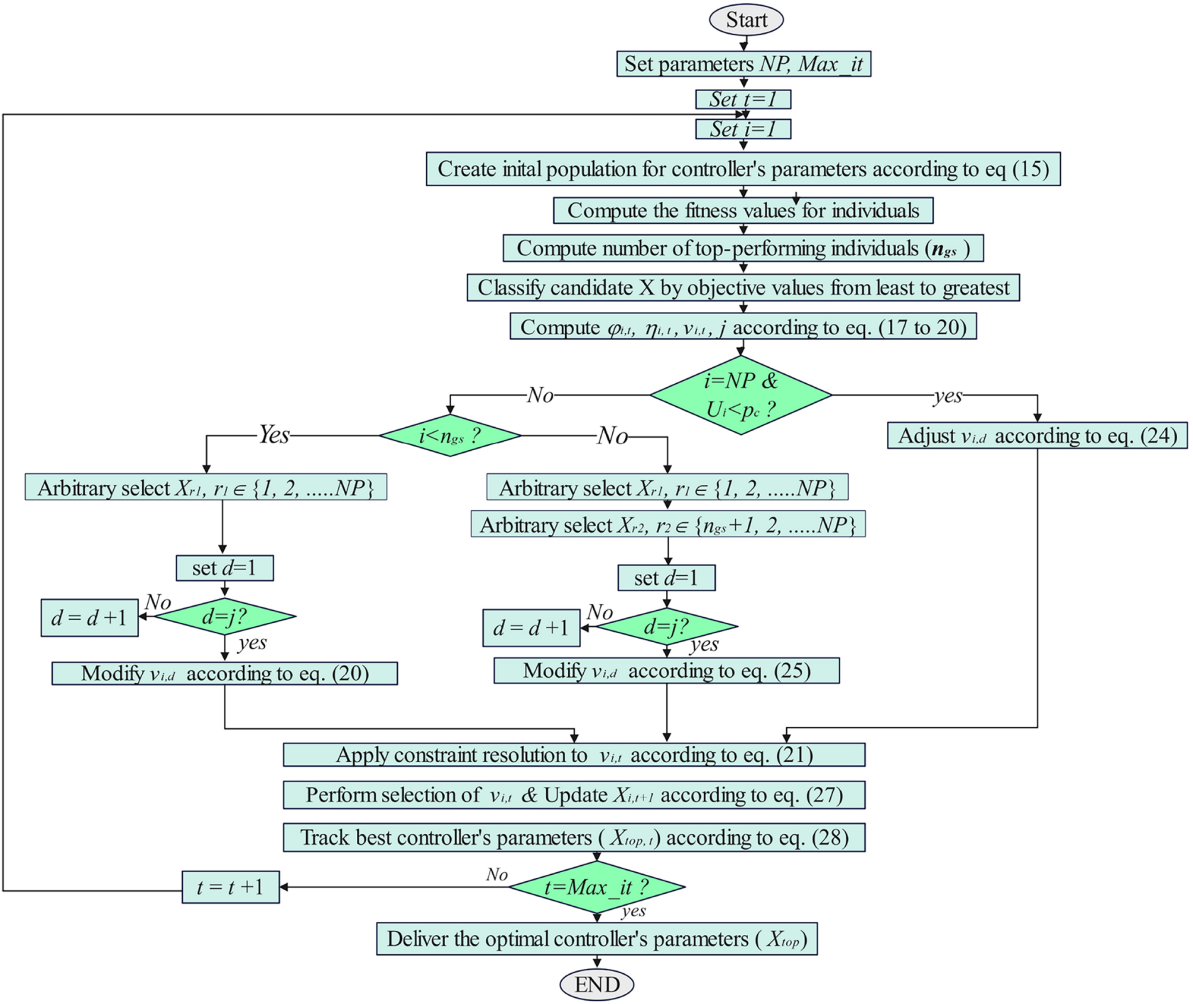
Flowchart for the proposed DCS optimization algorithm.

In DCS, algorism starts by creating a group of answers initialized with arbitrary values. It then iteratively searches for effective solutions, adjusting each solution’s position based on exploration and exploitation strategies. DCS employs a distinct Knowledge-gathering approach to ensure equilibrium these search processes. The optimization procedure ends following the predefined Requirements are fulfilled. The procedures of proposed DCS algorithm can be summarized in the flowchart in Figure 5^16^.

## Controller architecture

### Controller structure and challenge identification

This part discusses the implementation in the latest proposed controller FOPI-TID^µ^-PIDA tunning with DCS optimization For integrated voltage and frequency regulation loops (including secondary control loop that controller was exist with additional copy to the controller in AVR control loop) Configured for a Tri-region power system incorporating diesel thermal and reheat units per Region, together with an independent sustainable generation system.(PV soler cell, wind and hydro units).FOPI-TID^µ^-PIDA is cascaded proposed controller for three series regulators. Each one has various benefits like FOPI enhanced robustness, flexibility, and increased withstanding to parameter changes and outside disturbances, its characteristic Equations are ${C}_{1}\left(s\right)$ (31). While TID^μ^ in ${C}_{2}\left(s\right)$ (32) refers to Tilt-Integral-Derivative control, constructed by regeneration of PID traditional version (Proportional-Integral-Derivative) control. the Feature of a fractional order in μ make the integral and derivative actions are taken to a non-integer (fractional) power, providing better adaptability to complex system dynamics. The TID^μ^ regulator is properly high in controlling systems that require fine-tuning beyond traditional PID methods due to time delays or higher-order dynamics that cannot be adequately modeled using simple integer-order controllers. ${C}_{3}\left(s\right)$ expand the characteristic Equation for PIDA (33). ${C}_{4}\left(s\right)$ (34) implement total transfer function to the cascaded regulators ${C}_{1}\left(s\right)$, ${C}_{2}\left(s\right)$ and ${C}_{4}\left(s\right)$. This controller performance was tested in this model at section V with full discussion, outcome result was compared to other three controller that led to prove with evidence superiority of proposed controller. Another comparison performed to proposed controller based on DCS optimization technique with other algorisms AEO, DO and Rung Kutta, that exemplification the reliability and resilience of the suggested DCS optimization.

31 $${C}_{1}\left(s\right)=KP+\frac{KI}{{s}^{\lambda }}$$

32 $${C}_{2}\left(s\right)=\frac{KT}{{s}^\frac{1}{n}}+\frac{KI2}{s}+KD1\left[\frac{N1}{1+\frac{N1}{{s}^{\mu }}}\right]$$

33 $${C}_{3}\left(s\right)=KP2+\frac{KI3}{s}+KD2\left[\frac{N2\cdot s}{s+N2}\right]+KD2\cdot KD3\left[\frac{N2.N3.{s}^{2}}{\left(s+N2\right).\left(s+N3\right)}\right]$$

34 $${C}_{4}\left(s\right)={C}_{1}\left(s\right)\cdot {C}_{2}\left(s\right)\cdot {C}_{3}\left(s\right)$$

### Controller philosophy and cost function

**Fig. 6.**
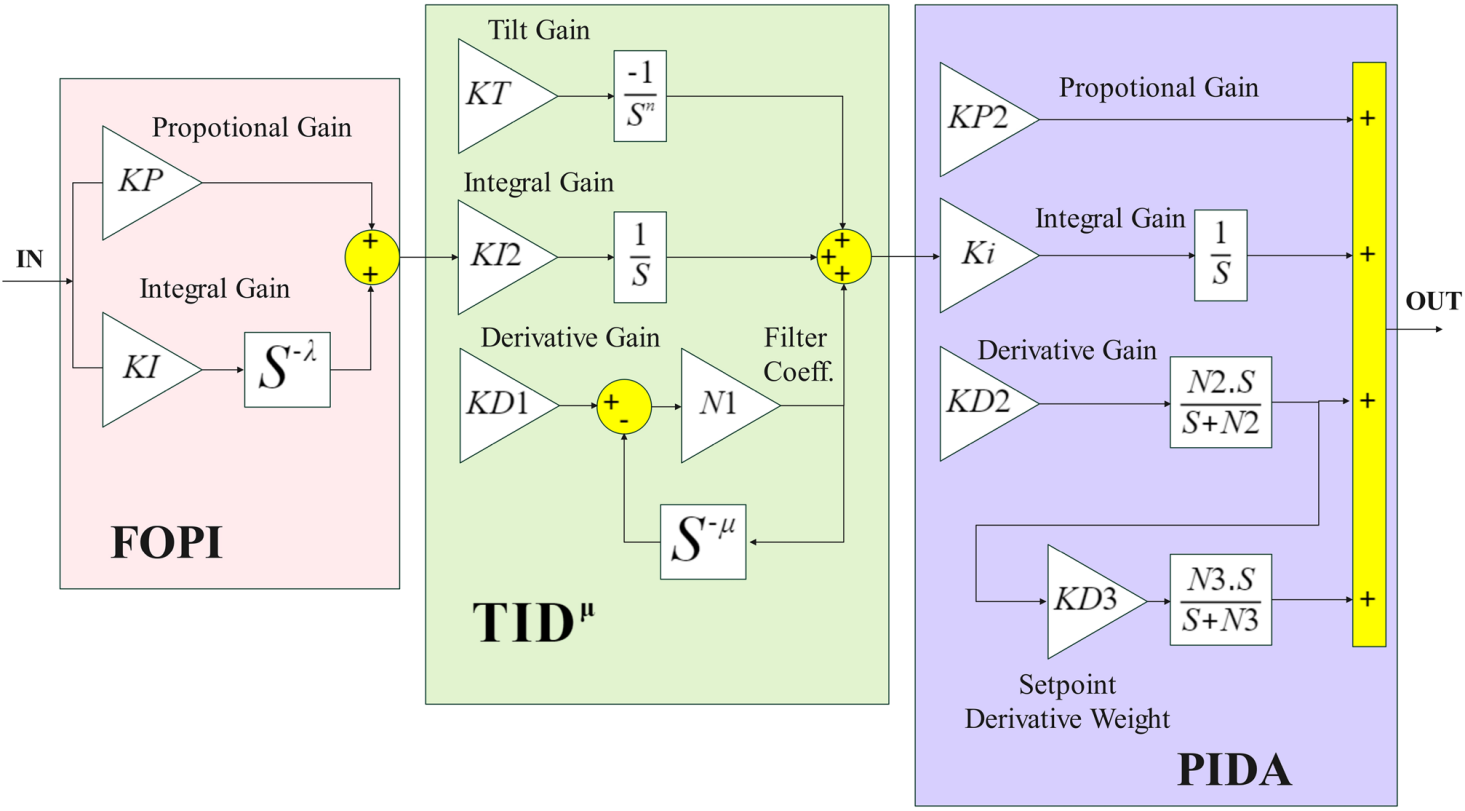
The combined controller structure.

The principal and strategy for the proposed controller structure design to combine each regulator with own benefits in one form that maximize the ability of generated proposed controller by exploitation advantage of each independent regulator. Figure 6 represent structure of the proposed controller including cascaded three regulators. Goal beyond for this strategy is to lessen frequency deviation ($\Delta {f}_{y}$) and lower tie-line power deviations ($\Delta {P}_{tie xy}$), Due to Variabilities within the system, in the LFC loop, and refine the dynamic voltage response across three areas by reducing voltage deviations ($\Delta {V}_{x}$). to get the desired performance -–probable fitness function ITAE is used with probably setting the parameter for the proposed controller. In this analysis, the integral time absolute error, generally called ITAE, was selected as the fitness function to assess the controller’s performance, as defined by Equations (35) and (36)^19^. ITAE is predicted to yield the best approach for substantially Controlling transient deviations and shortening settling time in the integrated LFC-AVR system. Block diagram for the proposed control strategy is illustrated in Figure 6^29,43^.

35 $$ITAE={\int }_{0}^{T}t\cdot \left[|\Delta {f}_{x}|+|\Delta {V}_{x}|+|\Delta {P}_{tie xy}|\right] dt$$

Here, $\Delta {\mathrm{f}}_{\mathrm{k}}$ and $\Delta {\mathrm{V}}_{\mathrm{k}}$ are the frequency deviation and voltage deviation values in $x$ ^th^ area, $\Delta {\mathrm{P}}_{\text{tie xy}}$ Indicates the discrepancy in the tie-line between regions $x$ and $y$. where $T$ Symbolizes the simulation time, with the following limitations applied to the controller parameters:

36 $$\begin{array}{c}0\le KP\le 1\\ 0\le KI\le 1\\ 0\le \lambda \le 1\\ 0\le KT\le 5\\ 0\le KI2\le 5\\ 0\le KD1\le 1\\ 1\le n\le 10\\ 100\le N1\le 500\\ 0\le \mu \le 1\\ \begin{array}{c}0\le KP2\le 5\\ 0\le ki\le 1\\ \begin{array}{c}0\le KD2\le 1\\ \begin{array}{c}0\le KD3\le 1\\ \begin{array}{c}100\le N2\le 500\\ 100\le N3\le 500\end{array}\end{array}\end{array}\end{array}\end{array}$$

## Simulation results and discussions

This section demonstrates the outcome findings of using different recent optimization algorithms for fine tuning the key parameters of the proposed FOPI-TIDµ-PIDA regulator. The computational effort required for tuning the FOPI-TIDμ-PIDA controller was evaluated based on the performance of the optimization algorithms employed. All controller parameters were tuned offline, allowing the design to avoid any online real-time computational overhead. The evaluation was performed using MATLAB R2023a on a system equipped with an Intel Core i7 processor @ 2.60 GHz and 32 GB of RAM. Each metaheuristic algorithm was executed with the same number of search agents (30) and iterations (100), resulting in 3,000 function evaluations per run. The execution times for the four optimizers were approximately: AEO (45.3 s), DO (47.6 s), RUN (43.8 s), and DCS (46.1 s). Among them, DCS provided the best convergence behavior while maintaining a competitive computational load. Although the FOPI-TIDμ-PIDA structure involves 15 parameters, the optimization algorithms efficiently explored the solution space within a reasonable time, validating the feasibility of implementing the proposed control structure in practice. Since the entire optimization was handled offline, it poses no delay or complexity during real-time system operation.

For more details, this section has been divided into the following 3 subsections.

### Comparisons between the diverse optimization strategies implemented for tunning the proposed regulator (FOPI-TID^µ^-PIDA)

**Fig. 7:**
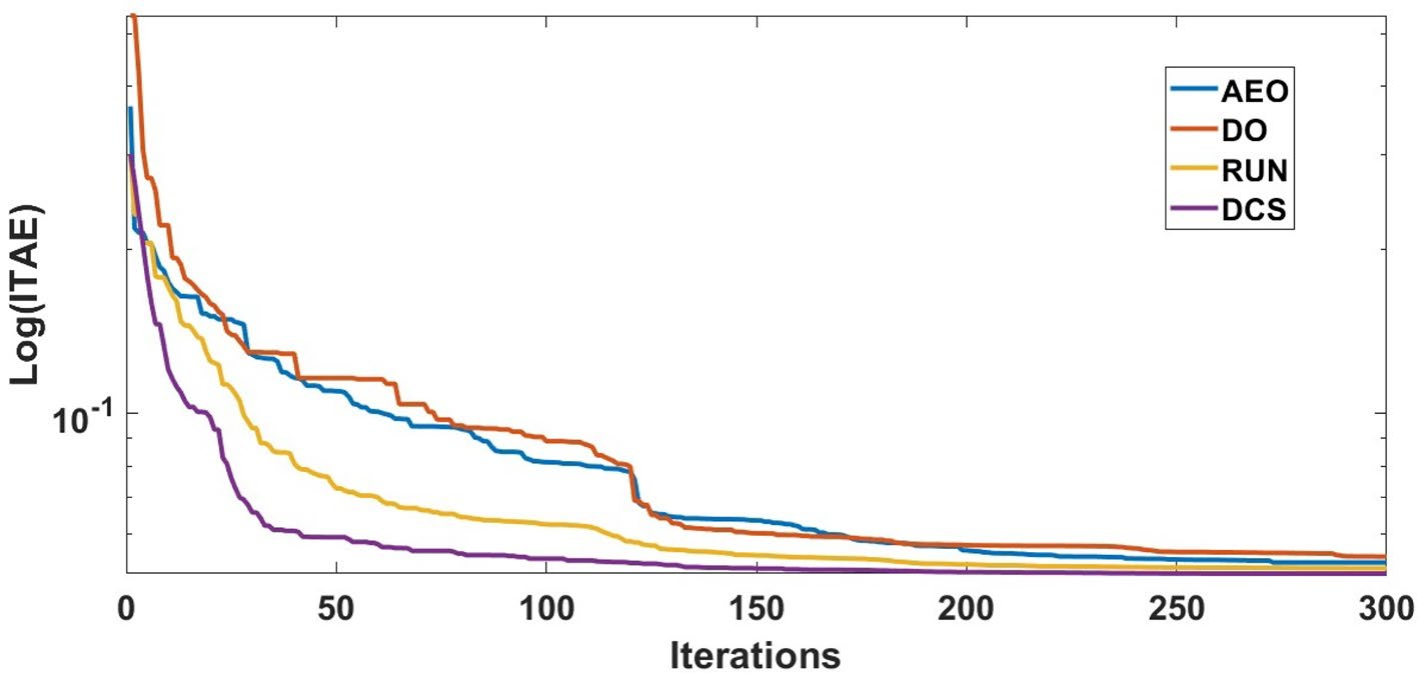
The convergence curve comparison for the different optimization techniques.

**Fig. 8:**
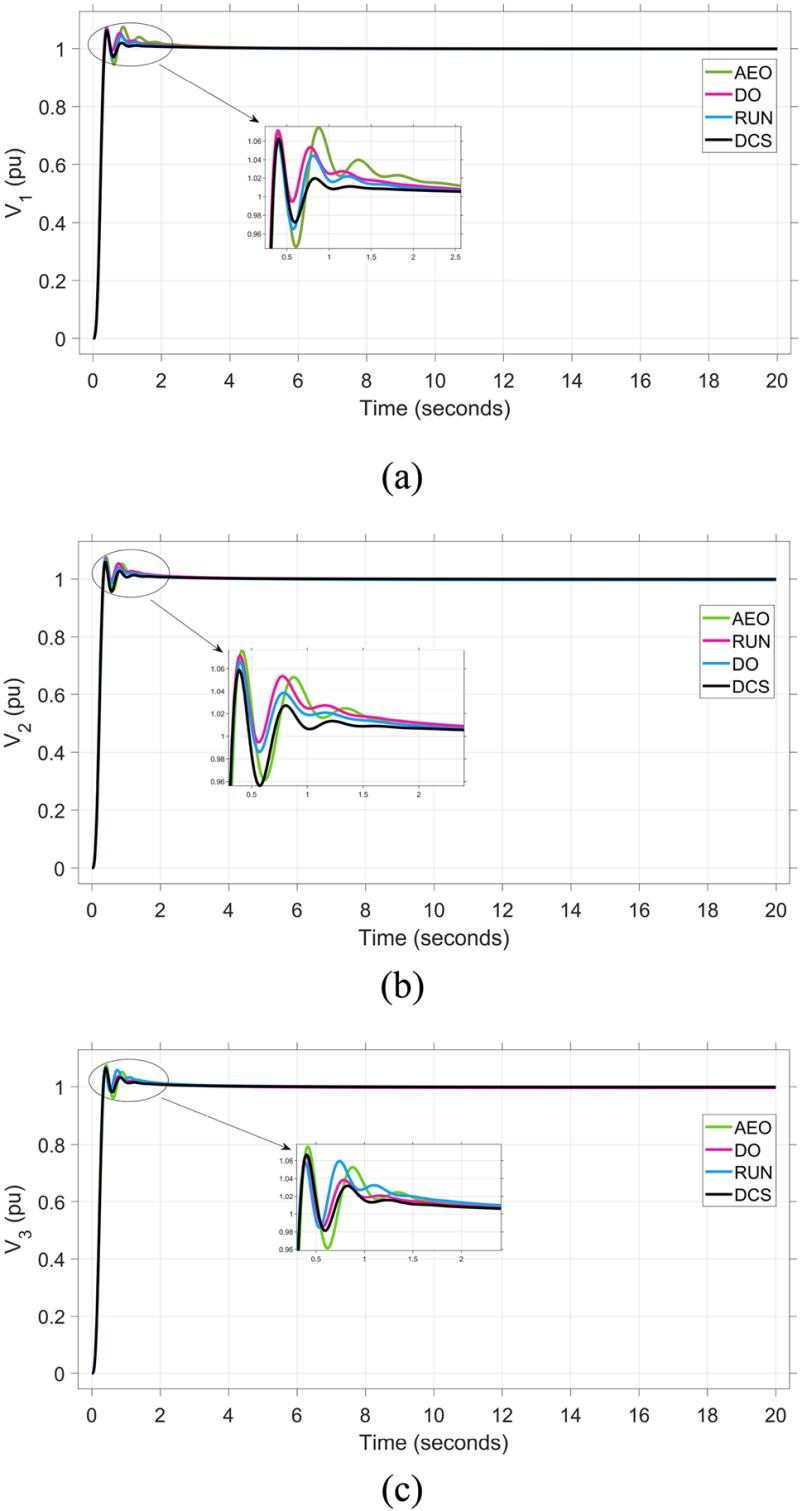
The voltage performance of the triple-area system when using different optimization techniques for tuning the proposed controller: (**a**) V_1_, (**b**) V_2_, and (**c**) V_3_.

**Fig. 9:**
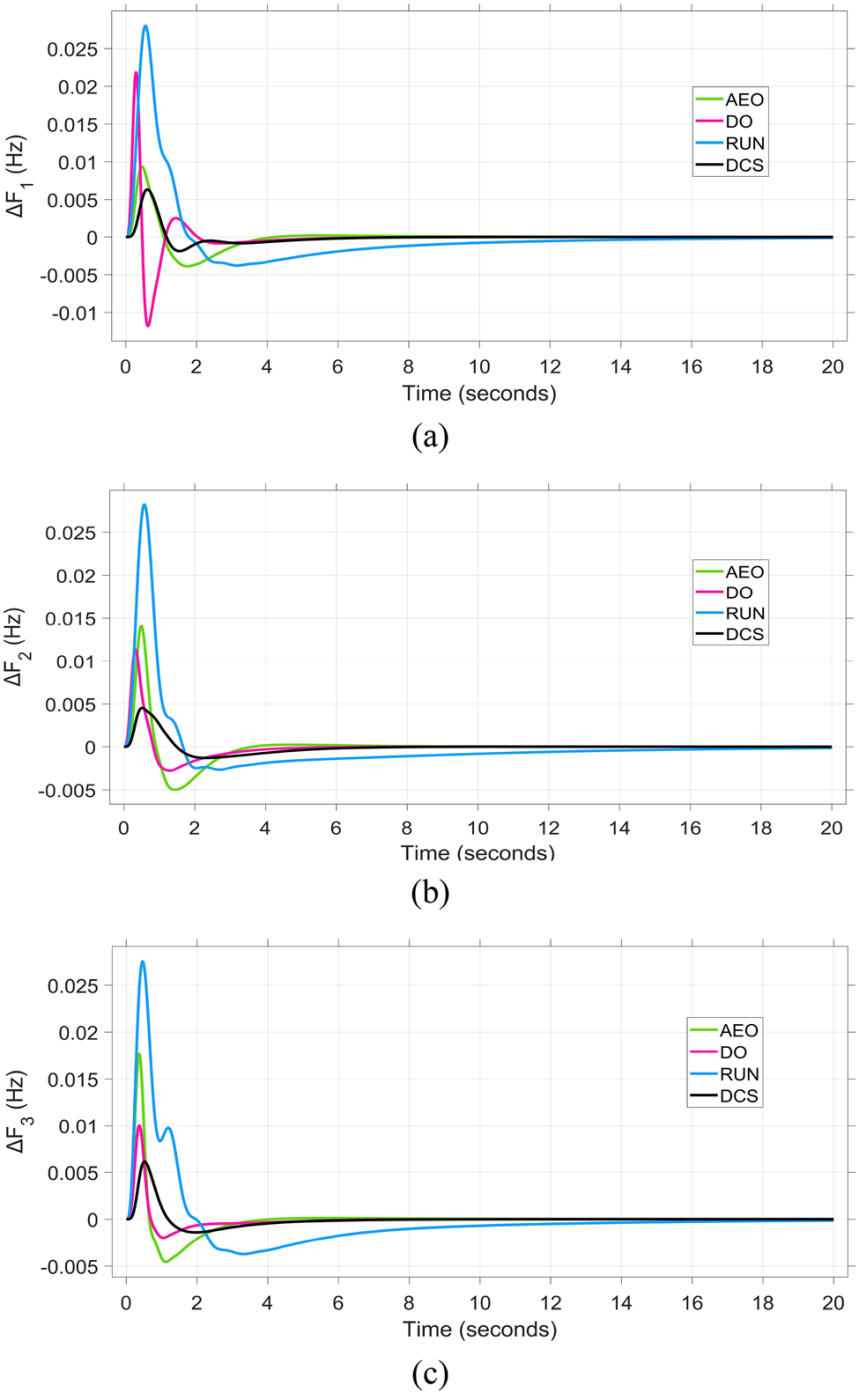
The frequency deviation performance to triple-area when using various optimization algorism for tuning the proposed controller: (**a**)∆F_1_, (**b**)∆F_2_, and (**c**)∆F_3_.

**Fig. 10:**
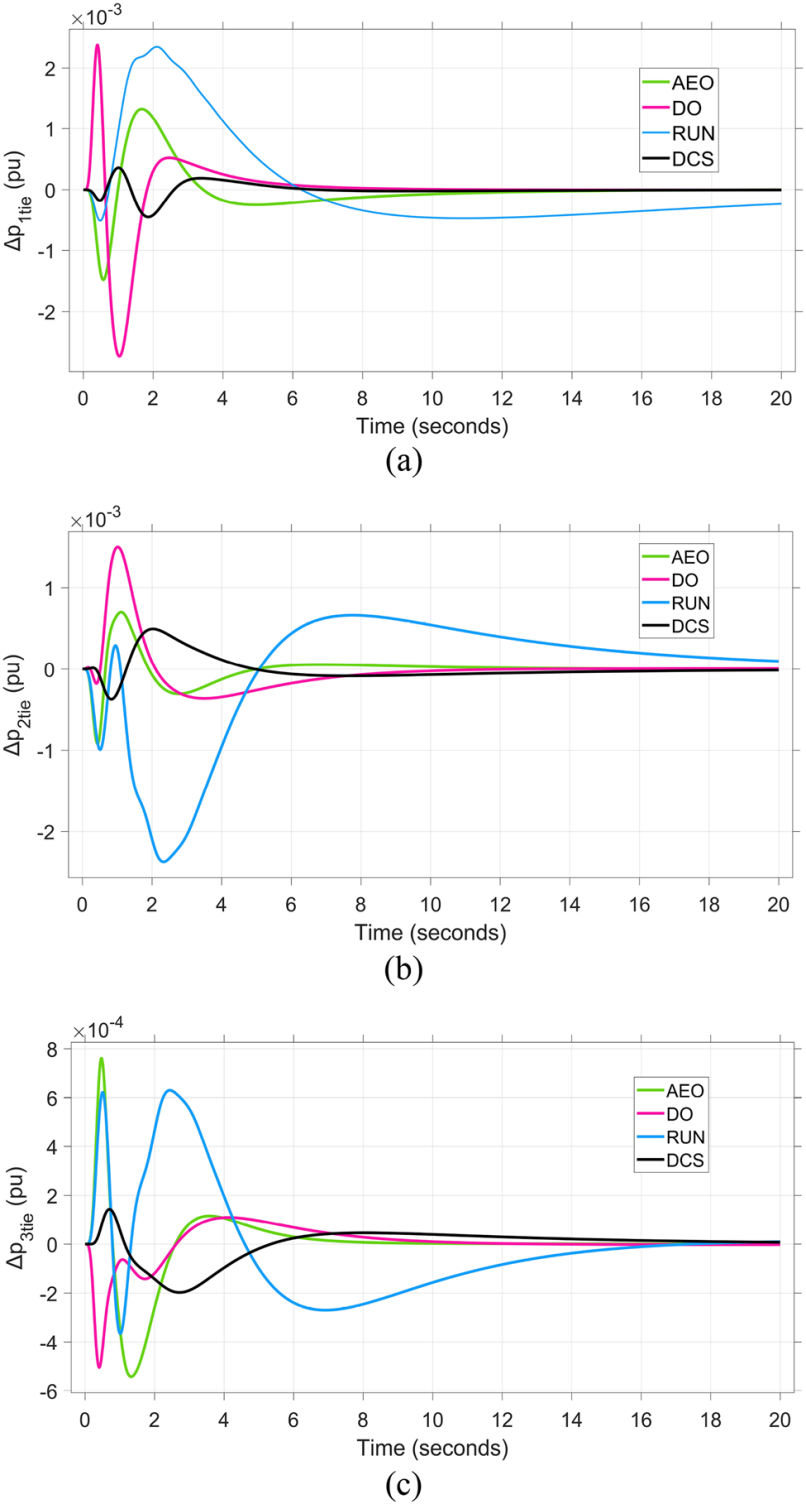
The fluctuation tie-line power performance to triple-area system when using different optimization techniques for tuning the suggested regulator: (**a**)∆P_1tie_, (**b**)∆P_2tie_, and (**c**)∆P_3tie_.

In this section, competency of the proposed DCS algorithm is verified according to study of both load frequency control (LFC) and automatic voltage regulator (AVR). The validation of the DCS The performance and efficiency of DCS optimization are examined by contrasting it with different optimization algorithms such as AEO, DO and RUN. The comparison is conducted by enhancing the designed controller`s parameters to improve both voltage and frequency stability in the considered triple-interconnected power system supplied by diverse energy providers. The parameters of FOPI-TID^µ^-PIDA controller at each concerned optimization algorithm are summarized in Table 1. Accordingly, the dynamic response of the proposed controller under different optimization algorithms is illustrated in Table 2. Comparison between the convergency curves of all considered algorithms is shown in Figure 7, which indicates the superiority of the proposed DCS. By evaluating the effectiveness of the DCS strategy against AEO, DO and RUN, The DCS technique can be validated to possess improved stabilization over other techniques. The dynamic behavior for system under different algorithms is depicted in Figures 8, 9, and 10. To sum up, the proposed regulator, refined using the DCS approach, demonstrates superior performance in minimizing peak overshoot (POS) that does not exceed 1.057, peak deviation undershoots (PUS), settling time (${T}_{st}$) that does not exceed 7.5 msec, and the Integral time absolute error (ITAE) equals 0.0507, which is considered as the objective function.

**Table 1:** Parameter setting of the proposed controller when using multiple optimization techniques.

**Algorism**	**Coefficient**		**Area 1**	**Area 2**	**Area 3**
**AEO**	**LFC**	${\boldsymbol{K}}{\boldsymbol{P}}$	0.836	0.681	0.518
		${\boldsymbol{K}}{\boldsymbol{I}}$	0.871	0.939	0.898
		${\boldsymbol{\lambda}}$	0.252	0.564	0.644
		${\boldsymbol{K}}{\boldsymbol{T}}$	4.883	3.055	2.166
		${\boldsymbol{K}}{\boldsymbol{I}}2$	1.246	3.364	4.010
		${\boldsymbol{K}}{\boldsymbol{D}}1$	0.344	0.605	0.311
		${\boldsymbol{n}}$	4.861	7.934	7.140
		${\boldsymbol{N}}1$	444	440	446
		${\boldsymbol{\mu}}$	0.380	0.711	0.354
		${\boldsymbol{K}}{\boldsymbol{P}}2$	3.976	3.795	4.919
		${\boldsymbol{k}}{\boldsymbol{i}}$	0.195	0.513	0.898
		${\boldsymbol{K}}{\boldsymbol{D}}2$	0.676	0.554	0.479
		${\boldsymbol{K}}{\boldsymbol{D}}3$	0.012	0.063	0.037
		${\boldsymbol{N}}2$	400	349	178
		${\boldsymbol{N}}3$	251	265	219
	**AVR**	${\boldsymbol{K}}{\boldsymbol{P}}$	0.991	0.978	0.978
		${\boldsymbol{K}}{\boldsymbol{I}}$	0.419	0.342	0.024
		${\boldsymbol{\lambda}}$	0.189	0.106	0.134
		${\boldsymbol{K}}{\boldsymbol{T}}$	4.883	3.426	4.897
		${\boldsymbol{K}}{\boldsymbol{I}}2$	0.126	0.011	0.002
		${\boldsymbol{K}}{\boldsymbol{D}}1$	0.297	0.712	0.724
		${\boldsymbol{n}}$	6.934	9.528	9.903
		${\boldsymbol{N}}1$	464	497	339
		${\boldsymbol{\mu}}$	0.799	0.947	0.751
		${\boldsymbol{K}}{\boldsymbol{P}}2$	2.313	4.007	4.799
		${\boldsymbol{k}}{\boldsymbol{i}}$	0.011	0.096	0.115
		${\boldsymbol{K}}{\boldsymbol{D}}2$	0.639	0.909	0.640
		${\boldsymbol{K}}{\boldsymbol{D}}3$	0.014	0.011	0.022
		${\boldsymbol{N}}2$	465	481	458
		${\boldsymbol{N}}3$	461	276	474
**DO**	**LFC**	${\boldsymbol{K}}{\boldsymbol{P}}$	0.284	0.196	0.978
		${\boldsymbol{K}}{\boldsymbol{I}}$	0.932	0.743	0.376
		${\boldsymbol{\lambda}}$	0.414	0.462	0.895
		${\boldsymbol{K}}{\boldsymbol{T}}$	3.271	1.557	3.474
		${\boldsymbol{K}}{\boldsymbol{I}}2$	4.283	4.744	2.002
		${\boldsymbol{K}}{\boldsymbol{D}}1$	0.468	0.571	0.405
		${\boldsymbol{n}}$	6.668	1.924	7.228
		${\boldsymbol{N}}1$	164	194	150
		${\boldsymbol{\mu}}$	0.663	0.285	0.571
		${\boldsymbol{K}}{\boldsymbol{P}}2$	0.903	1.835	2.675
		${\boldsymbol{k}}{\boldsymbol{i}}$	0.565	0.058	0.952
		${\boldsymbol{K}}{\boldsymbol{D}}2$	0.546	0.582	0.107
		${\boldsymbol{K}}{\boldsymbol{D}}3$	0.006	0.001	0.021
		${\boldsymbol{N}}2$	270	152	173
		${\boldsymbol{N}}3$	499	162	412
	**AVR**	${\boldsymbol{K}}{\boldsymbol{P}}$	0.284	0.196	0.978
		${\boldsymbol{K}}{\boldsymbol{I}}$	0.306	0.003	0.395
		${\boldsymbol{\lambda}}$	0.104	0.520	0.101
		${\boldsymbol{K}}{\boldsymbol{T}}$	4.852	4.983	4.932
		${\boldsymbol{K}}{\boldsymbol{I}}2$	0.113	0.023	0.001
		${\boldsymbol{K}}{\boldsymbol{D}}1$	0.168	0.559	0.556
		${\boldsymbol{n}}$	7.752	9.981	9.979
		${\boldsymbol{N}}1$	494	498	494
		${\boldsymbol{\mu}}$	0.770	0.751	0.775
		${\boldsymbol{K}}{\boldsymbol{P}}2$	3.376	3.532	4.934
		${\boldsymbol{k}}{\boldsymbol{i}}$	0.025	0.117	0.148
		${\boldsymbol{K}}{\boldsymbol{D}}2$	0.865	0.572	0.830
		${\boldsymbol{K}}{\boldsymbol{D}}3$	0.034	0.019	0.018
		${\boldsymbol{N}}2$	498	500	500
		${\boldsymbol{N}}3$	500	500	498
**RUN**	**LFC**	${\boldsymbol{K}}{\boldsymbol{P}}$	0.388	0.253	0.529
		${\boldsymbol{K}}{\boldsymbol{I}}$	0.652	0.539	0.249
		${\boldsymbol{\lambda}}$	0.682	0.419	0.783
		${\boldsymbol{K}}{\boldsymbol{T}}$	4.041	1.121	2.847
		${\boldsymbol{K}}{\boldsymbol{I}}2$	4.893	4.269	1.926
		${\boldsymbol{K}}{\boldsymbol{D}}1$	0.332	0.449	0.362
		${\boldsymbol{n}}$	8.934	1.726	5.679
		${\boldsymbol{N}}1$	480	289	303
		${\boldsymbol{\mu}}$	0.670	0.211	0.426
		${\boldsymbol{K}}{\boldsymbol{P}}2$	0.783	1.269	1.129
		${\boldsymbol{k}}{\boldsymbol{i}}$	0.439	0.027	0.449
		${\boldsymbol{K}}{\boldsymbol{D}}2$	0.836	0.367	0.259
		${\boldsymbol{K}}{\boldsymbol{D}}3$	0.002	0.001	0.001
		${\boldsymbol{N}}2$	460	152	428
		${\boldsymbol{N}}3$	426	429	367
	**AVR**	${\boldsymbol{K}}{\boldsymbol{P}}$	0.812	0.729	0.693
		${\boldsymbol{K}}{\boldsymbol{I}}$	0.259	0.149	0.426
		${\boldsymbol{\lambda}}$	0.081	0.065	0.011
		${\boldsymbol{K}}{\boldsymbol{T}}$	7.936	6.170	9.275
		${\boldsymbol{K}}{\boldsymbol{I}}2$	0.186	0.153	0.289
		${\boldsymbol{K}}{\boldsymbol{D}}1$	0.279	0.126	0.329
		${\boldsymbol{n}}$	9.619	8.179	9.991
		${\boldsymbol{N}}1$	242	500	409
		${\boldsymbol{\mu}}$	0.672	0.327	0.563
		${\boldsymbol{K}}{\boldsymbol{P}}2$	4.925	3.169	1.171
		${\boldsymbol{k}}{\boldsymbol{i}}$	0.011	0.041	0.036
		${\boldsymbol{K}}{\boldsymbol{D}}2$	0.726	0.982	0.682
		${\boldsymbol{K}}{\boldsymbol{D}}3$	0.031	0.078	0.014
		${\boldsymbol{N}}2$	367	459	409
		${\boldsymbol{N}}3$	469	500	500
**DCS**	**LFC**	${\boldsymbol{K}}{\boldsymbol{P}}$	0.997	0.973	0.998
		${\boldsymbol{K}}{\boldsymbol{I}}$	0.001	0.998	0.001
		${\boldsymbol{\lambda}}$	0.101	0.104	0.499
		${\boldsymbol{K}}{\boldsymbol{T}}$	4.988	4.999	5
		${\boldsymbol{K}}{\boldsymbol{I}}2$	4.991	0.002	5
		${\boldsymbol{K}}{\boldsymbol{D}}1$	0.983	1	0.976
		${\boldsymbol{n}}$	3.098	3.823	7.440
		${\boldsymbol{N}}1$	499	150	500
		${\boldsymbol{\mu}}$	0.996	0.1	0.1
		${\boldsymbol{K}}{\boldsymbol{P}}2$	4.998	4.876	5
		${\boldsymbol{k}}{\boldsymbol{i}}$	0.001	0.565	0
		${\boldsymbol{K}}{\boldsymbol{D}}2$	0.999	1	0.930
		${\boldsymbol{K}}{\boldsymbol{D}}3$	0.0001	0.055	0.103
		${\boldsymbol{N}}2$	500	500	500
		${\boldsymbol{N}}3$	247	500	500
	**AVR**	${\boldsymbol{K}}{\boldsymbol{P}}$	0.983	0.996	0.998
		${\boldsymbol{K}}{\boldsymbol{I}}$	0.002	0	0.998
		${\boldsymbol{\lambda}}$	0.101	0.104	0.992
		${\boldsymbol{K}}{\boldsymbol{T}}$	4.994	4.997	0.108
		${\boldsymbol{K}}{\boldsymbol{I}}2$	0.003	0.001	3.181
		${\boldsymbol{K}}{\boldsymbol{D}}1$	0.210	1	0.215
		${\boldsymbol{n}}$	9.978	10	10
		${\boldsymbol{N}}1$	500	407	189
		${\boldsymbol{\mu}}$	0.997	0.717	0.721
		${\boldsymbol{K}}{\boldsymbol{P}}2$	4.879	4.991	4.999
		${\boldsymbol{k}}{\boldsymbol{i}}$	0.115	0.129	0.054
		${\boldsymbol{K}}{\boldsymbol{D}}2$	0.993	0.553	1
		${\boldsymbol{K}}{\boldsymbol{D}}3$	0.011	0.023	0.034
		${\boldsymbol{N}}2$	500	498	500
		${\boldsymbol{N}}3$	492	500	469

**Table 2:** The dynamic performance of the studied model when using multiple optimization techniques with the proposed controller.

**Controller**	**Optimization**	**Feature**	**V**_**1(pu)**_	**V**_**2(pu)**_	**V**_**3(pu)**_	**ΔF**_**1(Hz)**_	**ΔF**_**2(Hz)**_	**ΔF**_**3(Hz)**_	**ΔP**_**tie1(pu)**_	**ΔP**_**tie2(pu)**_	**ΔP**_**tie3(pu)**_	**ITAE**
**FOPI-TID**^**µ**^**-PIDA** **(Proposed)**	**AEO**	**POS**	1.074	1.075	1.075	0.009	0.014	0.017	0.001	7 x10^−4^	7.6 x10^−4^	0.0706
		**PUS**	0.94	0.96	0.961	−0.003	−0.005	−0.004	−1.5x10^−3^	−9 x10^−4^	−5.4x10^−4^	
		**Tst**	4	3.5	3	8.5	8	8	12	12	9	
	**DO**	**POS**	1.071	1.065	1.066	0.021	0.011	0.01	0.023	1.5 x10^−3^	1.1 x10^−4^	0.0789
		**PUS**	0.99	0.986	0.986	−0.011	−0.0027	−0.002	−2.7x10^−3^	−3.6 x10^−4^	−5 x10^−4^	
		**Tst**	3.5	3.5	3	6.5	7	4.5	10	14	11	
	**RUN**	**POS**	1.062	1.071	1.058	0.028	0.03	0.027	0.023	3 x10^−4^	6.3 x10^−4^	0.0649
		**PUS**	0.96	0.994	0.984	−0.004	−0.0026	−0.003	−5 x10^−4^	−2.3 x10^−3^	−3.6 x10^−4^	
		**Tst**	3.5	3.5	3	17	18	17	20	20	18	
	**DCS**	**POS**	1.042	1.058	1.057	0.006	0.004	0.006	3 x10^−4^	1 x10^−4^	1 x10^−4^	0.0507
		**PUS**	0.937	0.954	0.959	−0.001	−0.001	−0.0013	−4 x10^−4^	−3.7 x10^−4^	−1 x10^−4^	
		**Tst**	3.5	3.5	3	6.5	4	4	6	7.5	5.8

v

Figure 8 demonstrates the system’s reactions to (a) voltage fluctuation in Area-1 (V_1_(pu)), (b) voltage variation in Area-2 (V_2_(pu)), and (c) voltage fluctuation in Area-3 (V_3_(pu)), using different optimization algorithms. The iterative try and error performed to FOPI-TID^µ^-PIDA controller based on the DCS compared to other algorisms reach to get the efficient signal in AVR control loop to compensate insufficient in reactive power. The previous claim about the voltage response superiority achieved using the DCS algorithm is clearly demonstrated in Figure 8, where it yields the lowest overshoot across all three areas. Specifically, the voltage overshoots were limited to 1.058 pu in Area 1 as shown in Figure 8a, 1.058 pu in Area 2 as shown in Figure 8b, and 1.067 pu in Area 3 as illustrated in Figure 8c, highlighting the consistency and stability of the DCS-tuned regulator.

The frequency and tie-line power responses are further examined in Figures 9 and 10, respectively. For Area 2, the frequency deviation response ΔF₂ presented in Figure 9b shows that the RUN-based optimization produces the largest overshoot of 0.03 Hz, while the DCS-based controller achieves a significantly smaller overshoot of 0.004 Hz, indicating better damping and disturbance rejection capability.

Similarly, in Area 3, the tie-line power deviation ΔP_tie3_ illustrated in Figure 10c reveals that the DCS algorithm results in the minimum settling time of 5.8s, whereas the RUN optimization exhibits a substantially longer settling time of 18 s. The efficient signal produced from proposed controller tuned based on the DCS algorism in LFC control loop to compensate insufficient in active power. lead to perform regulation for power fluctuation and damping frequency oscillations in fast response, to preserve stability and synchronism to the power and frequency for integrated power system. For the suggested regulator tuned by the DCS algorism frequency and tie-line power discrepancy reaction shows the minimal stabilization time relative to the other three techniques. The analysis ended with demonstration of DCS algorism over to the defined compared algorisms. The DCS has improved the ITAE index relative to that achieved by the proposed regulator built upon AEO, DO and RUN algorithms with percentage of enhancement (28.187%, 35.741% and 21.879%, respectively).

### Comparisons between the different dcs-based controllers

In this section, the proposed optimization algorithm (i.e., DCS) is applied to fine tune the key parameters of other controllers under different six scenarios of load variation, and changing the operating conditions and compared to dynamic response parameters that are obtained in the last section, as follows:

- **Scenario 1:** Sudden load variation (SLV) introduced in Area 1.
- **Scenario 2:** Variable multi-step load in Area 1, coupled with a step load in Area 2.
- **Scenario 3:** Variable multi- stage load in Area 1 while a stochastic random load in Area 2.
- **Scenario 4:** Unpredictable multi- stage load in Area 1 with voltage reference variations in the three areas.
- **Scenario 5:** Different operating conditions.
- **Scenario 6:** Cyber-attack conditions in smart grid environment.

#### SCENARIO 1: Analysis robustness by applying sudden loaD 10% SLV introduced in area 1

In this part, the power system is tested beneath a 0.1 pu Sudden load variations (SLV) applied in region 1, incorporating sustainable energy sources (SES) in the connected areas. A PV unit in region 1, a wind unit in region 2, and hydro units in region 3. The SLV is simulated by disconnecting certain generators, representing sudden blackouts resulting from all generators going offline at the stations. The proposed controller is subjected to 300 iterations with 30 search agents during the optimization process, following the same procedure applied to the compared controllers (FOPI-PIDD^2^, TFOIDFF, and FOPI-PI) based on the DCS optimization technique. This process aims to optimize coefficient selection that enable the recommended controller to effectively handle various disturbances that power systems may encounter in real-world scenarios. The optimized parameters for the considered controllers using the proposed DCS optimization algorithm are summarized in Table 3. The proposed controller demonstrates its superiority by restoring system stability with a fast response, achieving steady-state conditions more efficiently than the other controllers. The efficiency of the FOPI-TID^µ^-PIDA regulator, tuned based on the DCS algorithm, is contrasted with the FOPI-PIDD^2^, TFOIDFF, and FOPI-PI controllers.

**Table 3:** The optimum parameters obtained when applying DCS algorithm for other literature controllers.

**Regulator**	**Coefficient**		**Area 1**	**Area 2**	**Area 3**
**FOPI-PIDD**^**2**^	**LFC**	${\boldsymbol{K}}{\boldsymbol{P}}1$ ${\boldsymbol{K}}{\boldsymbol{I}}1$ ${\boldsymbol{n}}$ ${\boldsymbol{K}}{\boldsymbol{P}}2$ ${\boldsymbol{K}}{\boldsymbol{I}}2$ ${\boldsymbol{K}}{\boldsymbol{D}}1$ ${\boldsymbol{K}}{\boldsymbol{D}}2$ ${\boldsymbol{N}}1$ ${\boldsymbol{N}}2$	4.092 4.340 1.094 2.606 1.940 1.225 0.037 330 235	2.608 0.547 2.375 2.065 2.751 0.026 0.070 150 184	3.472 0.463 2.640 2.240 1.494 0.311 0.088 500 162
	**AVR**	${\boldsymbol{K}}{\boldsymbol{P}}1$ ${\boldsymbol{K}}{\boldsymbol{I}}1$ ${\boldsymbol{n}}$ ${\boldsymbol{K}}{\boldsymbol{P}}2$ ${\boldsymbol{K}}{\boldsymbol{I}}2$ ${\boldsymbol{K}}{\boldsymbol{D}}1$ ${\boldsymbol{K}}{\boldsymbol{D}}2$ ${\boldsymbol{N}}1$ ${\boldsymbol{N}}2$	3.788 2.891 9.645 4.001 0.108 0.812 0.042 500 500	4.985 0.190 6.279 4.126 0.174 0.784 0.045 500 500	4.972 0.240 6.207 4.976 0.164 0.909 0.064 500 500
**TFOIDFF**	**LFC**	${\boldsymbol{K}}{\boldsymbol{T}}$ ${\boldsymbol{K}}{\boldsymbol{I}}$ ${\boldsymbol{K}}{\boldsymbol{D}}$ ${\boldsymbol{n}}$ ${\boldsymbol{\lambda}}1$ ${\boldsymbol{\mu}}$ $\boldsymbol{\rm N}$ ${\boldsymbol{\lambda}}2$	9.815 0.001 0.990 9.981 0.793 0.368 150 0.124	9.998 0.151 0 1.605 0.100 0.983 500 0.365	9.923 0.241 0.002 6.754 0.648 0.427 150 0.587
	**AVR**	${\boldsymbol{K}}{\boldsymbol{T}}$ ${\boldsymbol{K}}{\boldsymbol{I}}$ ${\boldsymbol{K}}{\boldsymbol{D}}$ ${\boldsymbol{n}}$ ${\boldsymbol{\lambda}}1$ ${\boldsymbol{\mu}}$ $\boldsymbol{\rm N}$ ${\boldsymbol{\lambda}}2$	0.716 1 0.879 0.918 0.985 0.446 219.914 0.212	2.358 0.996 0.927 10 0.937 0.311 500 0.532	2.195 0.709 0.837 10 0.928 0.335 150 0.506
**FOPI-PI**	**LFC**	${\boldsymbol{K}}{\boldsymbol{P}}1$ ${\boldsymbol{K}}{\boldsymbol{I}}1$ ${\boldsymbol{\lambda}}$ ${\boldsymbol{K}}{\boldsymbol{P}}2$ ${\boldsymbol{K}}{\boldsymbol{I}}2$	0.001 0.005 0.936 1.413 0.983	1 0.977 0.578 4.863 1	0.424 0.867 0.743 4.991 0.989
	**AVR**	${\boldsymbol{K}}{\boldsymbol{P}}1$ ${\boldsymbol{K}}{\boldsymbol{I}}1$ ${\boldsymbol{\lambda}}$ ${\boldsymbol{K}}{\boldsymbol{P}}2$ ${\boldsymbol{K}}{\boldsymbol{I}}2$	0.991 0.509 0.782 4.072 0.512	0.763 0.001 0.771 4.225 0.217	0.781 1 0.835 2.807 0.376

**Fig. 11:**
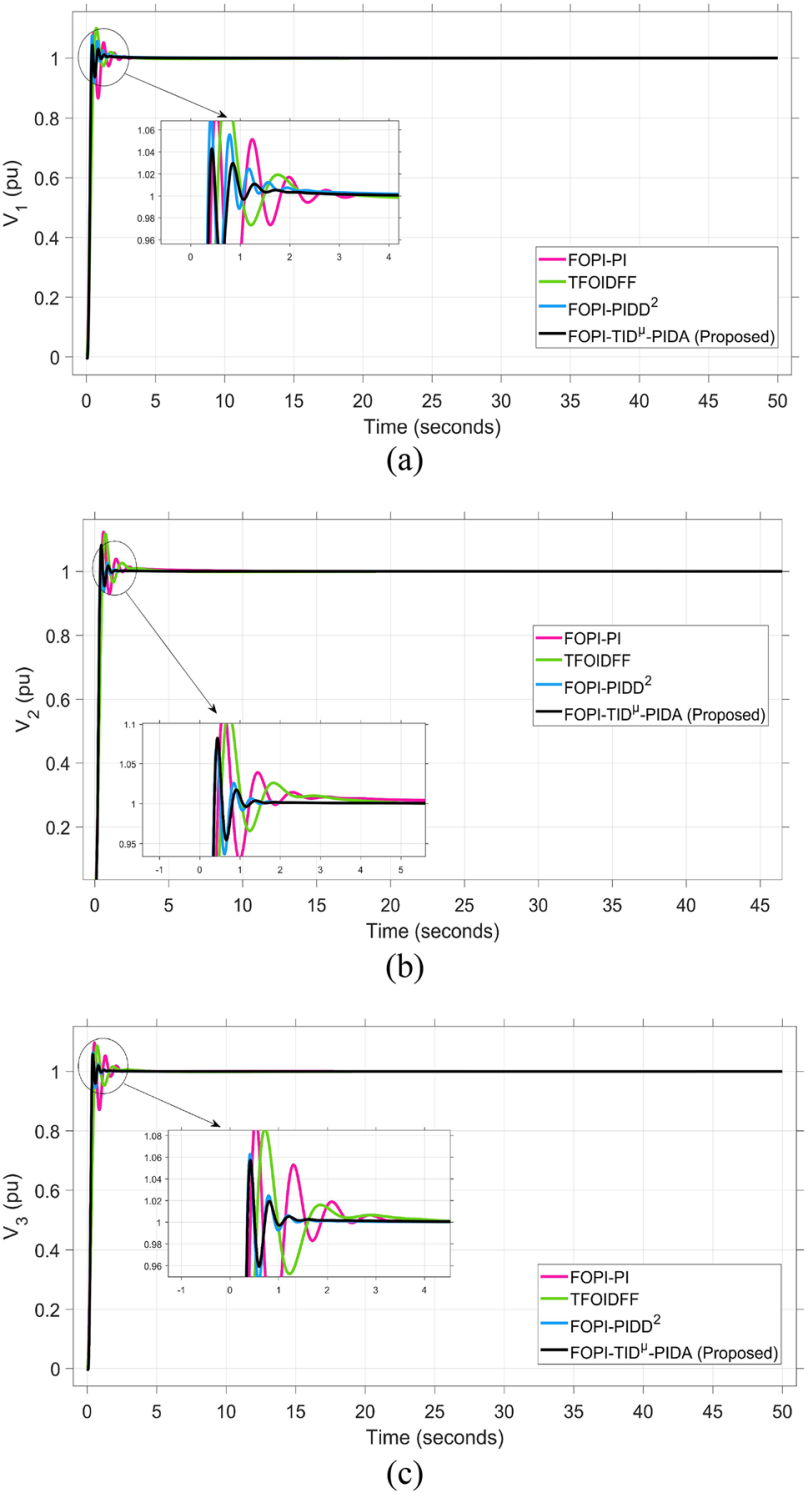
The voltage performance in Scenario 1 for the triple-area system when using different controllers tuned via DCS technique: (**a**) V_1_, (**b**) V_2_, and (**c**) V_3_.

**Fig. 12:**
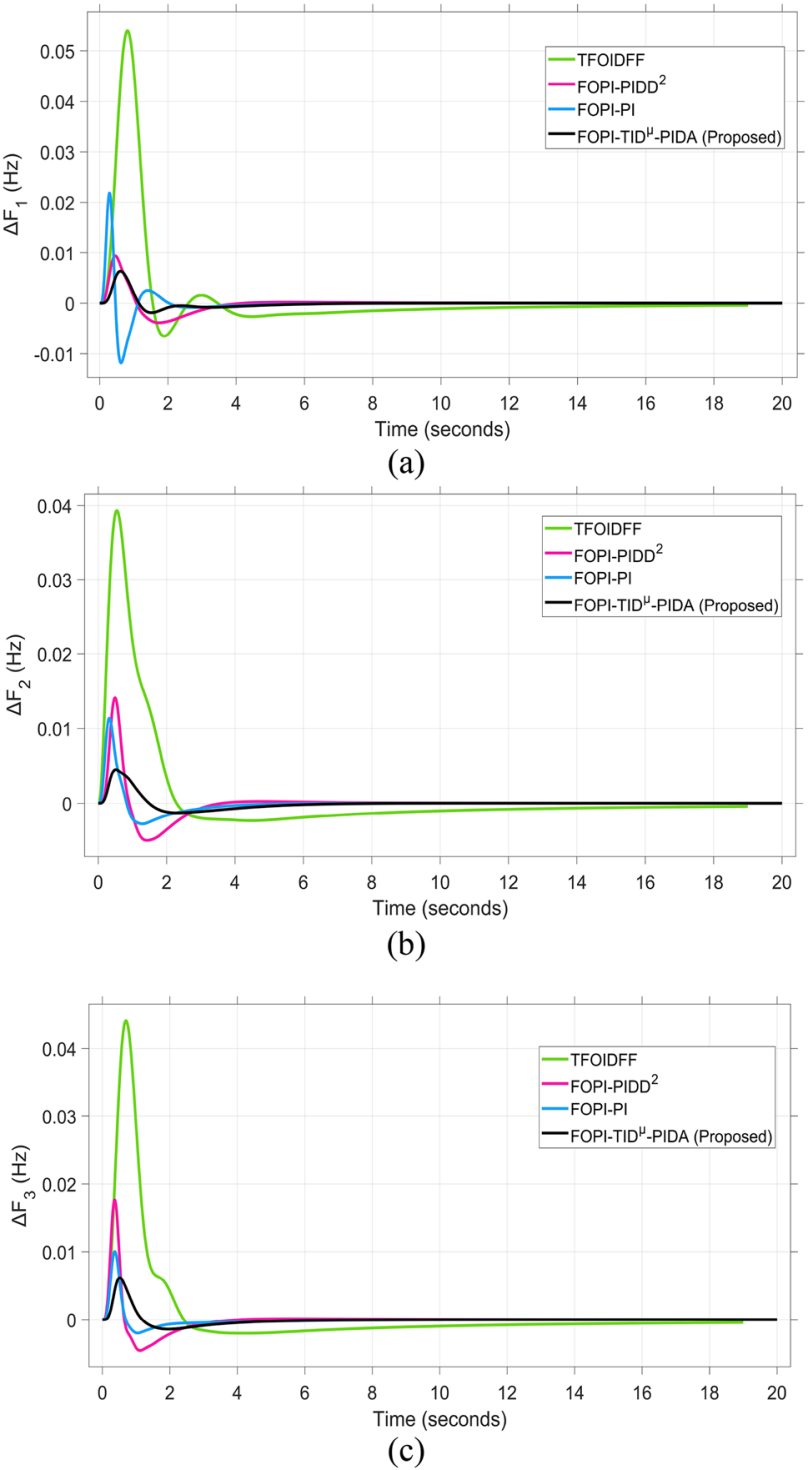
The effectiveness of frequency regulation in Scenario 1 for the triple-area model when using different controllers tuned via DCS technique: (**a**) ∆F_1_, (**b**) ∆F_2_, and (**c**) ∆F_3_.

**Fig. 13:**
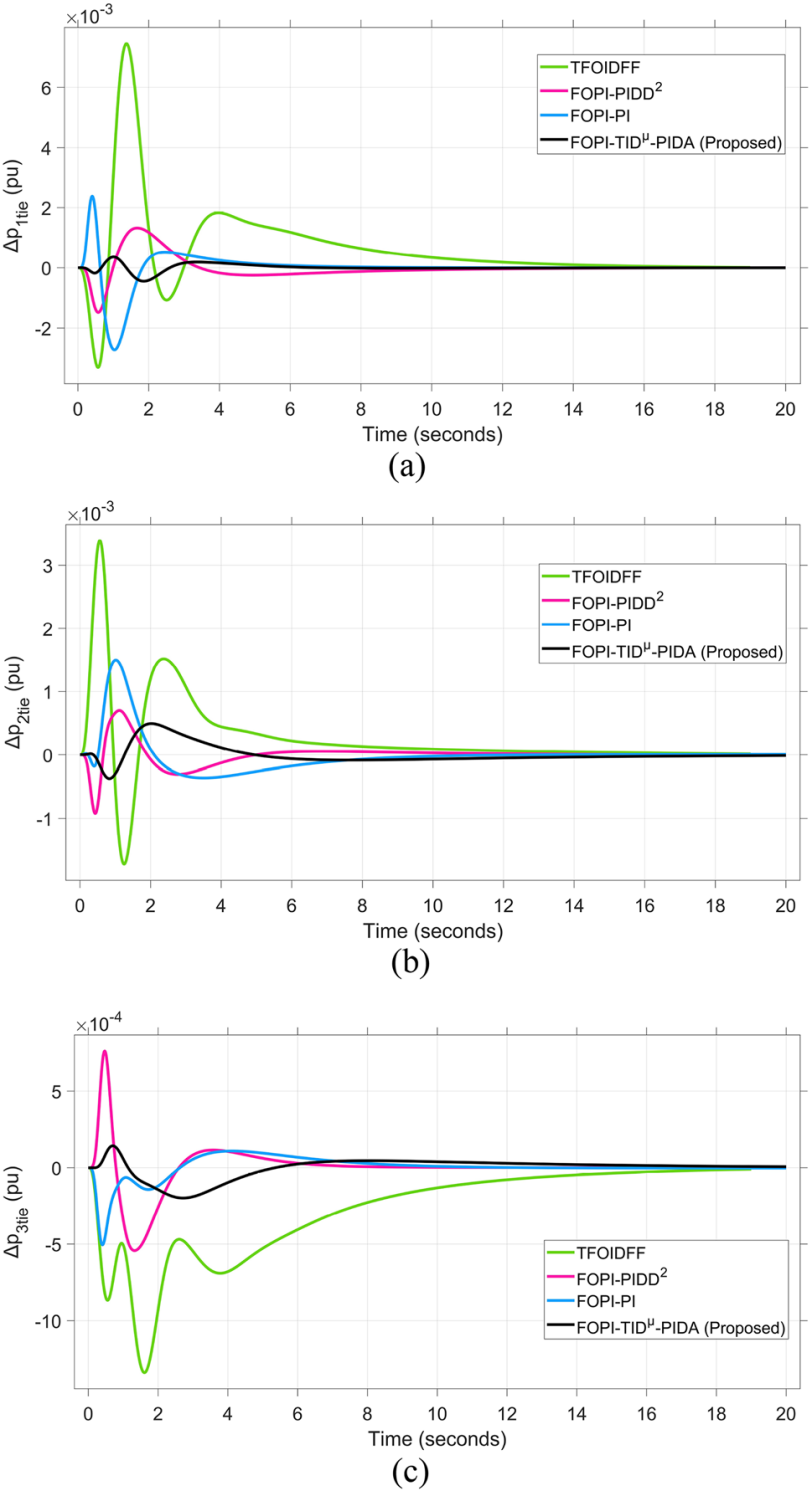
The fluctuation of tie-line power performance in Scenario 1 for the triple-area system when using different controllers tuned via DCS technique: (**a**) ∆P_1tie_, (**b**) ∆P_2tie_, and (**c**) ∆P_3tie_.

The system dynamic response such as voltage stability, frequency regulation, and tie-line power flow under this scenario is illustrated in Figures 10, 11, and 12, respectively showing that the proposed FOPI-TID^µ^-PIDA controller achieves significant reductions in undershoots, overshoots, and settling times. In Area 1, the voltage response V₁ (pu), shown in Figure 11a, indicates that the TFOIDFF controller records the longest settling time at approximately 14.4s, while the FOPI–TIDµ–PIDA controller achieves the fastest settling at 3.5 seconds, demonstrating superior dynamic recovery. The frequency deviation $\Delta F_{1}$ (Hz) in Figure 11a further supports this observation, where FOPI–TIDµ–PIDA exhibits the smallest overshoot, reaching only 6 × 10⁻^3^ Hz. In contrast, TFOIDFF experiences the highest transient peak, peaking around 0.54 Hz, reflecting a more aggressive but less stable response. In Area 2, $\Delta F2$ as indicated in Figure 12b, the system experiences a notable transient under TFOIDFF, with the output peaking at approximately 0.039 pu before returning to stability. For Area 3, the tie-line power deviation ΔP_tie3_, illustrated in Figure 13c, shows that TFOIDFF leads to the deepest undershoot, reaching −1×10⁻^3^ pu, highlighting its susceptibility to oscillatory behavior under abrupt load changes.

A comparison between the proposed FOPI-TID^µ^-PIDA controllers against other controllers under the fine tuning using the proposed DCS algorithm is summarized in Table 4 according to the dynamic response parameters of the considered system. The data show the superior performance of the FOPI-TID^µ^-PIDA regulator in managing the power system’s response to a 10% SLV in Area 1, with the contribution of SES in the connected areas. The controller ensures swift voltage regulation with fast error decay, effectively dampens frequency oscillations, and stabilizes tie-line power flow, all achieved through the DCS algorithm. The proposed controller outperforms the others in maintaining voltage and frequency stability and managing inter-area power flow, proving its effectiveness for practical power system applications. Additionally, the ITAE value of the FOPI-TID^µ^-PIDA controller, is significantly improved, the proposed controller designed based on DCS algorithm yielded the lowest objective function (0.0507), followed by the FOPI-PIDD^2^ (0.0689), then FOPI-PI (0.0713), lastly then TFOIDFF (0.1124). Improved by 26.415%, 28.892%, and 54.892% for the FOPI-PIDD^2^, FOPI-PI and TFOIDFF further validating its superior performance.

**Table 4:** The dynamic performance of the studied system when using different DCS-based controllers under the effect of Scenario 1.

**Controller**	**Features**	**V**_**1(pu)**_	**V**_**2(pu)**_	**V**_**3(pu)**_	**ΔF**_**1(Hz)**_	**ΔF**_**2(Hz)**_	**ΔF**_**3(Hz)**_	**ΔP**_**tie1(pu)**_	**ΔP**_**tie2(pu)**_	**ΔP**_**tie3(pu)**_	**ITAE**
**FOPI-PIDD**^**2**^	**POS**	1.075	1.072	1.062	9 x10^−3^	0.013	0.017	1 x10^−3^	6 x10^−4^	7 x10^−3^	0.0689
	**PUS**	0.920	0.936	0.944	−3 x10^−3^	−4 x10^−3^	−4 x10^−3^	−1 x10^−3^	−9 x10^−4^	−5 x10^−4^	
	**Tst**	11.4	4.2	3.2	7.5	8.4	6	10	12	10	
**TFOIDFF**	**POS**	1.1	1.117	1.086	0.054	0.039	0.04	7 x10^−3^	3 x10^−3^	0	0.1124
	**PUS**	0.93	0.965	0.952	−6 x10^−3^	−2 x10^−3^	−1.9 x10^−3^	−3 x10^−3^	−1 x10^−3^	−1 x10^−3^	
	**Tst**	14.4	5.3	4.5	15	16	14	16	12	16	
**FOPI-PI**	**POS**	1.803	1.123	1.095	0.021	0.011	9 x10^−3^	2 x10^−3^	1 x10^−3^	1.3 x10^−4^	0.0713
	**PUS**	0.866	0.930	0.87	−0.011	−2 x10^−3^	−1.9 x10^−3^	−2 x10^−3^	−3.6 x10^−4^	−5 x10^−5^	
	**Tst**	7.66	11	4	6.5	6.5	4	7.5	12	10	
**FOPI-TID**^**µ**^**-PIDA** **(Proposed)**	**POS**	1.042	1.058	1.057	0.006	0.004	0.006	3 x10^−4^	1 x10^−4^	1 x10^−4^	0.0507
	**PUS**	0.937	0.954	0.959	−0.001	−0.001	−0.0013	−4 x10^−4^	−3.7 x10^−4^	−1 x10^−4^	
	**Tst**	3.5	3.5	3	6.5	4	4	6	7.5	5.8	

#### SCENARIO 2: Applying variable multi-stage load in area 1 while considering sudden load in area 2

**Fig. 14:**
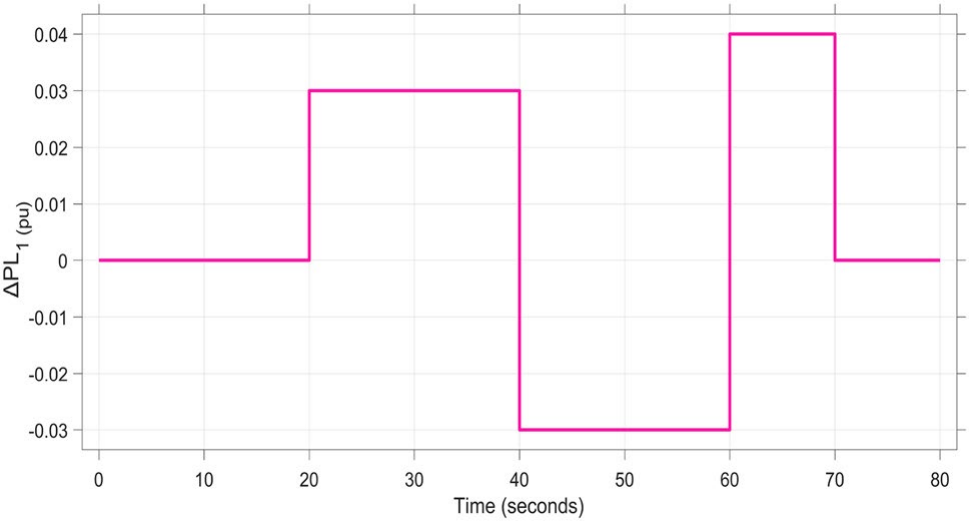
Multi-stage load disturbance profile.

**Fig. 15:**
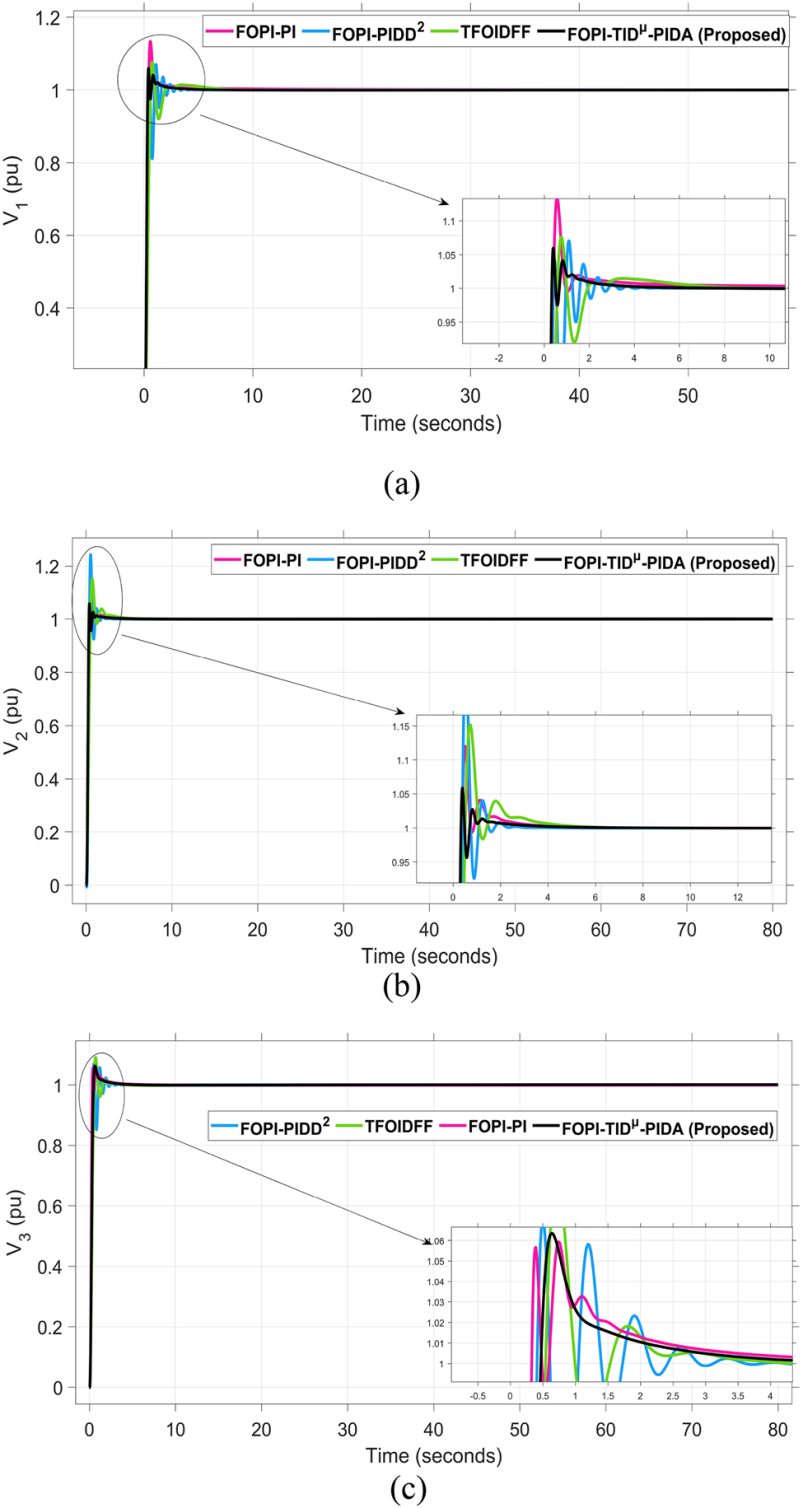
The voltage performance in Scenario 2 for the triple-area system when using different controllers tuned via DCS technique: (**a**) V_1_, (**b**) V_2_, and (**c**) V_3_.

In this subsection, evaluation of the dynamic performance of a hybrid Energy distribution network integrating Automatic Voltage Regulation (AVR) and Load Frequency Control (LFC) models under load fluctuation in two distinct areas is investigated. Sustainable energy sources (SES) are integrated as follows: a PV unit in region 1, a wind unit in region 2, and hydro units in region 3. As depicted in Figure 14, a variable multi-step load is utilized in region 1, while a sudden load variation is applied to Area 2. The system is tested using the proposed FOPI-TID^µ^-PIDA controller, which is compared to the other controllers based on the optimized fine-tuned parameters obtained in subsection B1 using the proposed DCS algorithm.Figure 15

**Fig. 16:**
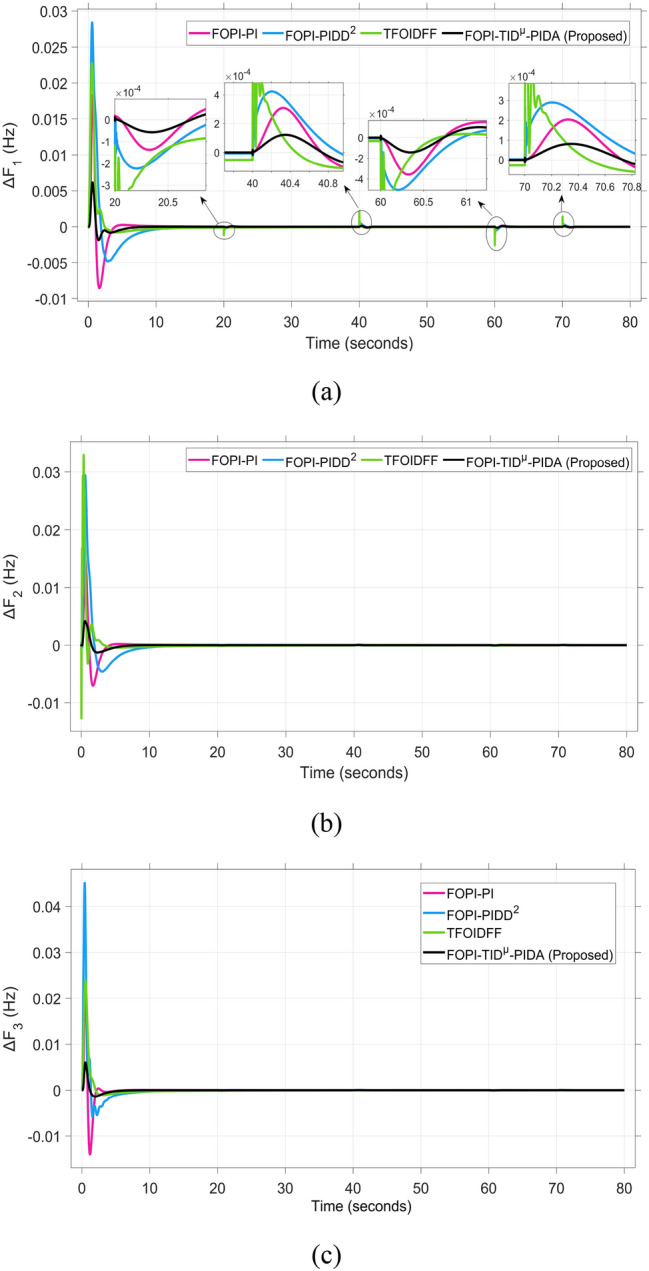
The effectiveness of frequency regulation in Scenario 2 for the triple-area model when using different controllers tuned via DCS technique: (**a**) ∆F_1_, (**b**) ∆F_2_, and (**c**) ∆F_3_.

**Fig. 17:**
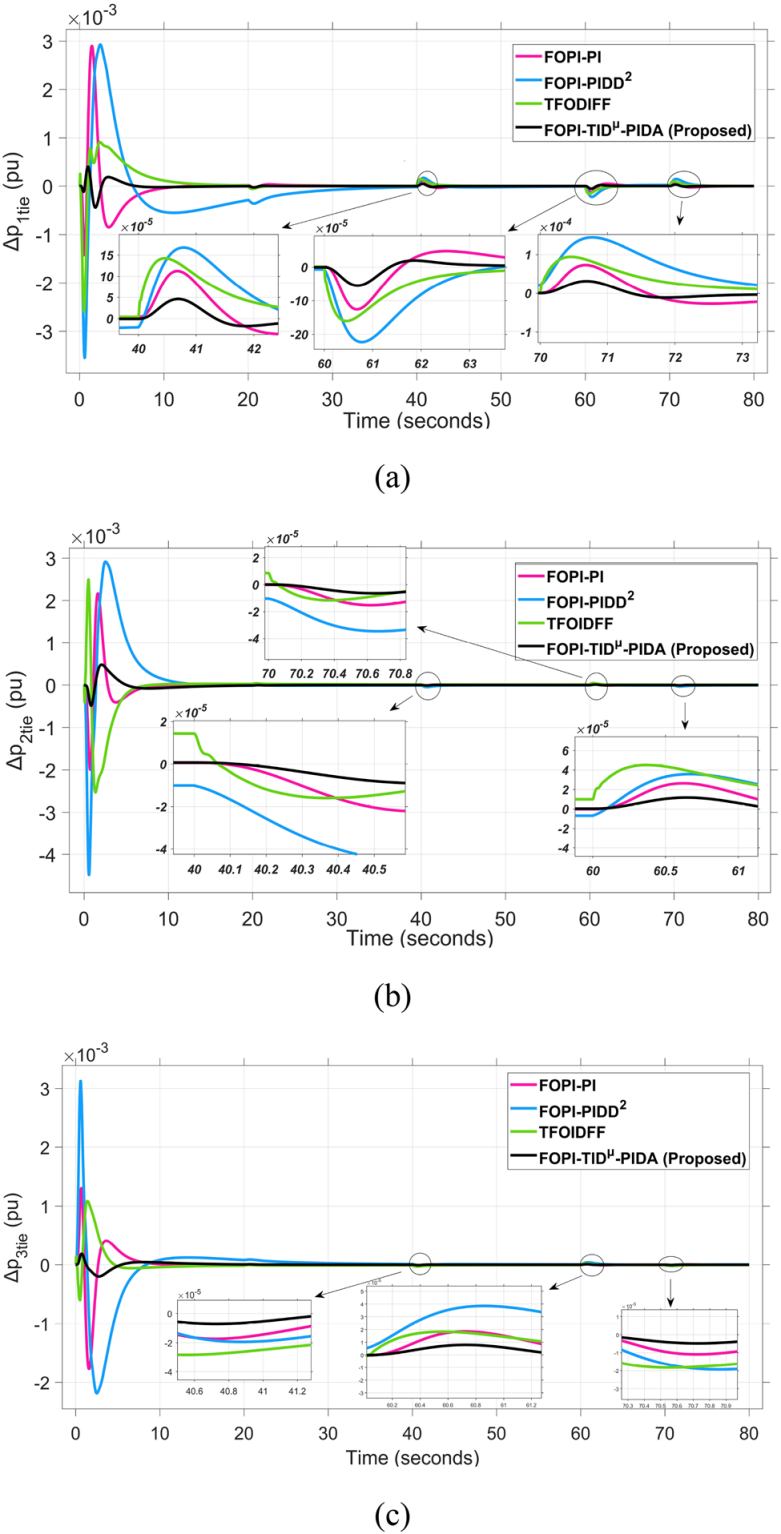
The fluctuation of tie-line power performance in Scenario 2 for the triple-area system when using different controllers tuned via DCS technique: (**a**) ∆P_1tie_, (**b**) ∆P_2tie_, and (**c**) ∆P_3tie_.

The resultant frequency deviations and tie-line power for all considered controllers are illustrated in Figures 16 and 17, respectively. From the figures, for Area 1, the frequency deviation $\Delta F_{1}$ reveals that the FOPI-PIDD^2^ controller produces the highest overshoot, reaching 0.028 Hz. Although during the third and fifth step load changes, the TFOIDFF controller exhibits sharp transient peaks, these do not exceed the maximum observed overshoot when compared with other control strategies in the same region. In Area 2, the voltage response V_2_ demonstrates that the FOPI-PIDD^2^ controller again yields the maximum overshoot, measured at 1.216 pu, under the impact of the sudden load event. As for Area 3, the tie-line power deviation ΔP_tie3_ confirms that the FOPI-PIDD^2^ controller also leads to the largest undershoot, reaching a value of approximately –2.1 × 10⁻^3^ pu, indicating a significant transient dip before stabilization. FOPI-TID^µ^-PIDA maintains the lowest undershoot, overshoot, and stabilization time for terminal voltage, synchronized frequency, and tie-line power flow, outperforming the other regulator in managing both sudden and gradual load variations. Accordingly, all considered parameters of dynamic response of the system for this scenario for all considered controllers are summarized in Table 5.

**Table 5:** The dynamic performance of the studied system when using different DCS-based controllers under the effect of Scenario 2.

**Controller**	**Features**	**V**_**1(pu)**_	**V**_**2(pu)**_	**V**_**3(pu)**_	**ΔF**_**1(Hz)**_	**ΔF**_**2(Hz)**_	**ΔF**_**3(Hz)**_	**ΔP**_**tie1(pu)**_	**ΔP**_**tie2(pu)**_	**ΔP**_**tie3(pu)**_
**FOPI-PIDD**^**2**^	**POS**	1.182	1.216	1.08	0.028	0.03	0.031	2.8 x10^−3^	2.8 x10^−3^	3.1 x10^−3^
	**PUS**	0.822	0.928	0.864	−3.8 x10^−3^	−2.6 x10^−3^	−0.004	9.3 x10^−3^	−4.2 x10^−3^	−2.1 x10^−3^
	**Tst**	8	9	6	18	18	19	36	28	24
**TFOIDFF**	**POS**	1.076	1.17	1.091	0.023	0.029	0.024	2.6 x10^−3^	2.6 x10^−3^	1 x10^−3^
	**PUS**	0.920	0.983	0.961	−8 x10^−3^	−3.1 x10^−3^	−0.001	−9 x10^−3^	−2.5 x10^−3^	−7 x10^−4^
	**Tst**	15	5	8	14	12	19	18	10	24
**FOPI-PI**	**POS**	1.133	1.120	1.06	0.018	0.015	0.023	0.003	2.1 x10^−3^	1.2 x10^−3^
	**PUS**	0.995	0.993	0.985	−8.5 x10^−3^	−7.2 x10^−3^	−0.014	−1.5 x10^−3^	−1.8 x10^−3^	−1.7 x10^−3^
	**Tst**	7	4	8	10	10	10	15	10	14
**FOPI-TID**^**µ**^**-PIDA** **(Proposed)**	**POS**	1.051	1.06	1.063	6.2 x10^−3^	4.3 x10^−3^	6 x10^−3^	3.9 x10^−4^	4 x10^−4^	1.5 x10^−4^
	**PUS**	0.970	0.957	0	−2 x10^−3^	−1.3 x10^−3^	−1.3 x10^−3^	−4 x10^−4^	−4 x10^−4^	−1.8 x10^−4^
	**Tst**	5	4	5	8	8	7	10	28	20

#### SCENARIO 3: Applying variable multi-stage load in area 1 while considering stochastic random load in area 2

**Fig. 18:**
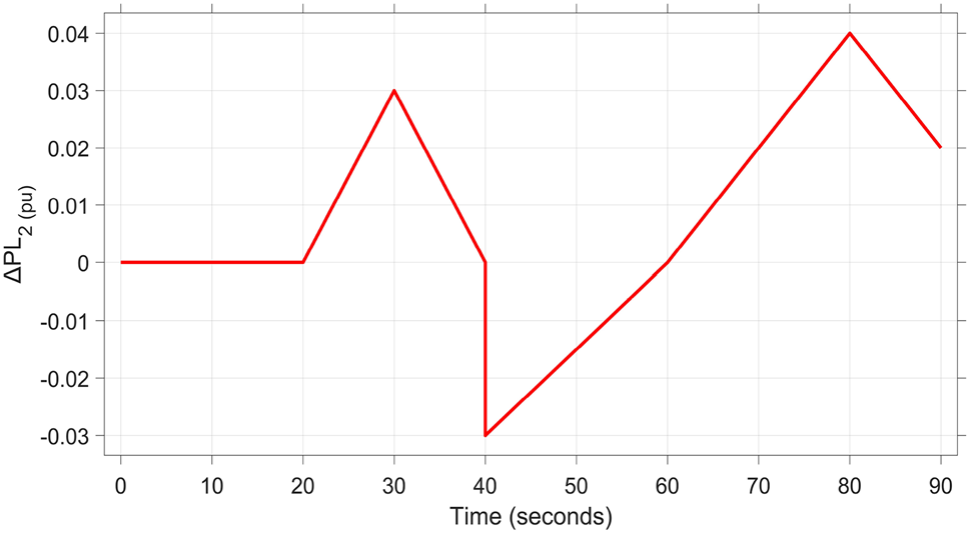
Stochastic random load profile.

In this subsection, further evaluation is conducted to indicate efficiency of the proposed FOPI-TIDµ-PIDA controller in handling specific types of disturbances. The Real-time performance of the hybrid power grid is tested under two distinct load disturbances, with the adoption of sustainable energy solutions (SES) in each region: a PV unit in region 1, a wind unit in region 2, and hydro units in region 3. The objective is to assess the impact of these combined disturbances on key performance metrics. A Multi-Stage Load (MSL) implemented in region 1, while a Stochastic Random Load (SRL) disturbance is applied to region 2 as shown in Figure 18. The SRL disturbance involves random variations in load levels, with each level maintained for random durations. This unpredictable load pattern challenges the system’s adaptability to sudden and irregular load changes.

**Fig. 19:**
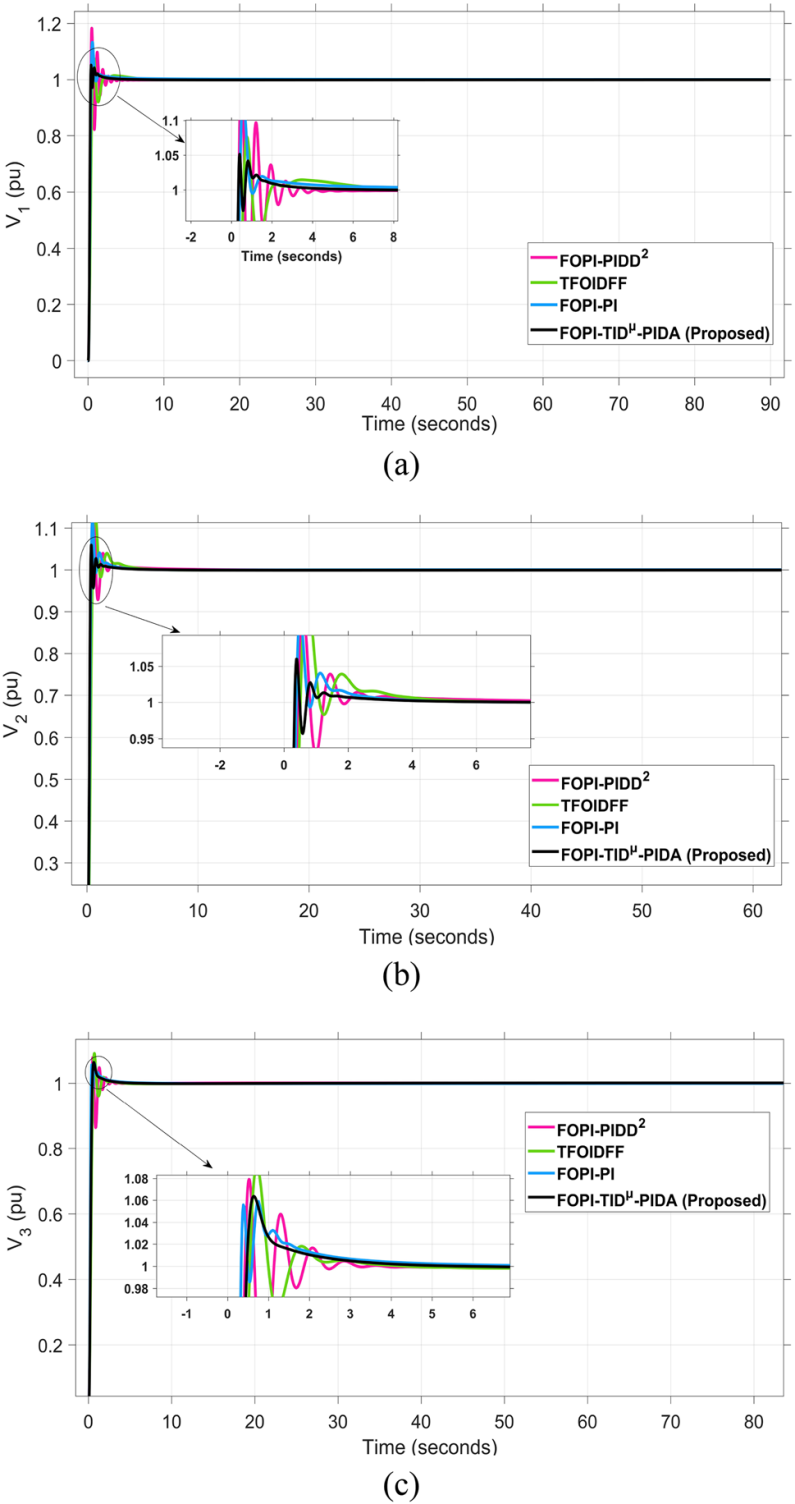
The voltage performance in Scenario 3 for the triple-area system when using different controllers tuned via DCS technique: (**a**) V_1_, (**b**) V_2_, and (**c**) V_3_.

**Fig. 20:**
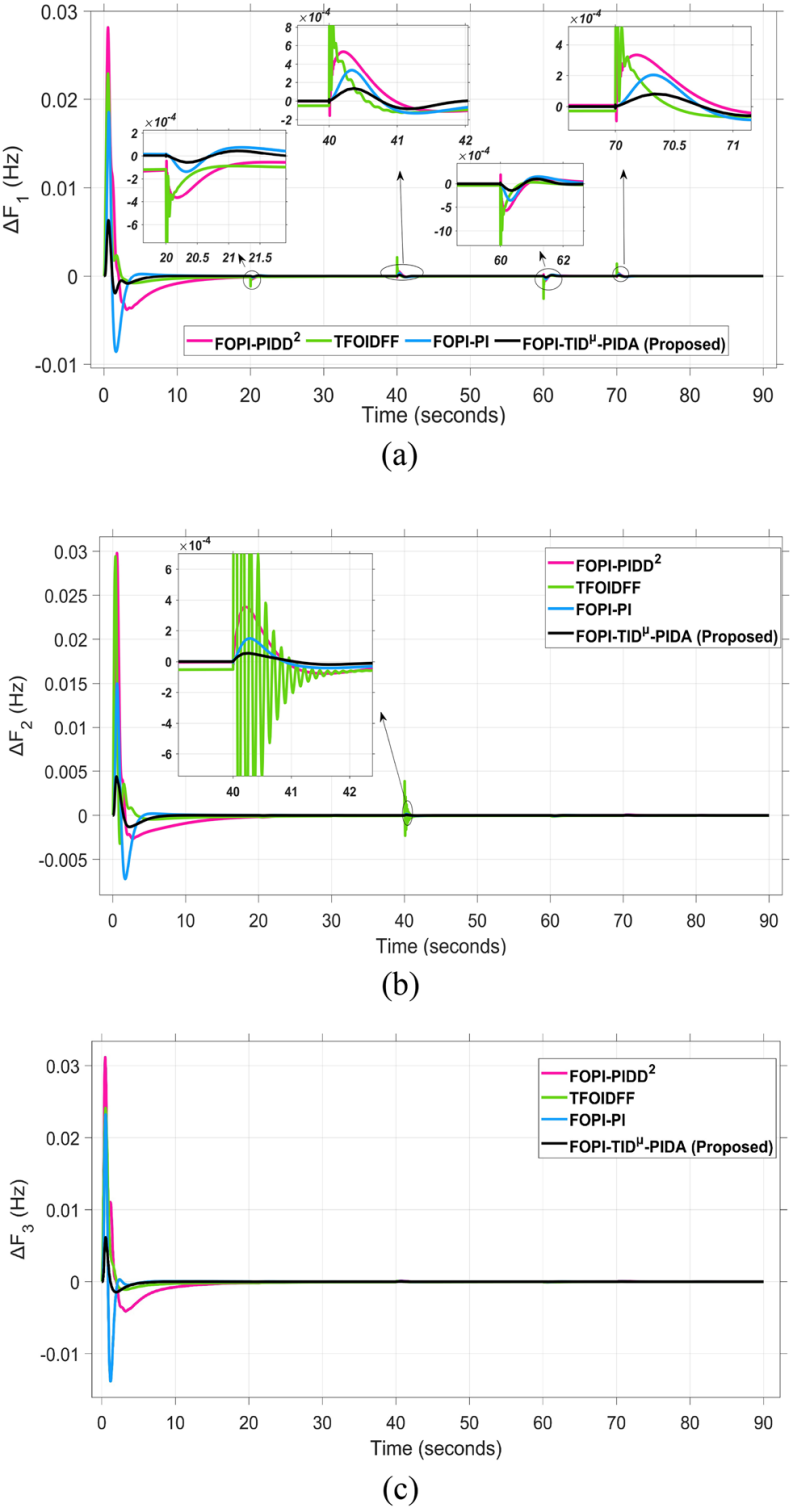
The effectiveness of frequency regulation in Scenario 3 for the triple-area model when using different controllers tuned via DCS technique: (**a**) ∆F_1_, (**b**) ∆F_2_, and (**c**) ∆F_3_.

**Fig. 21:**
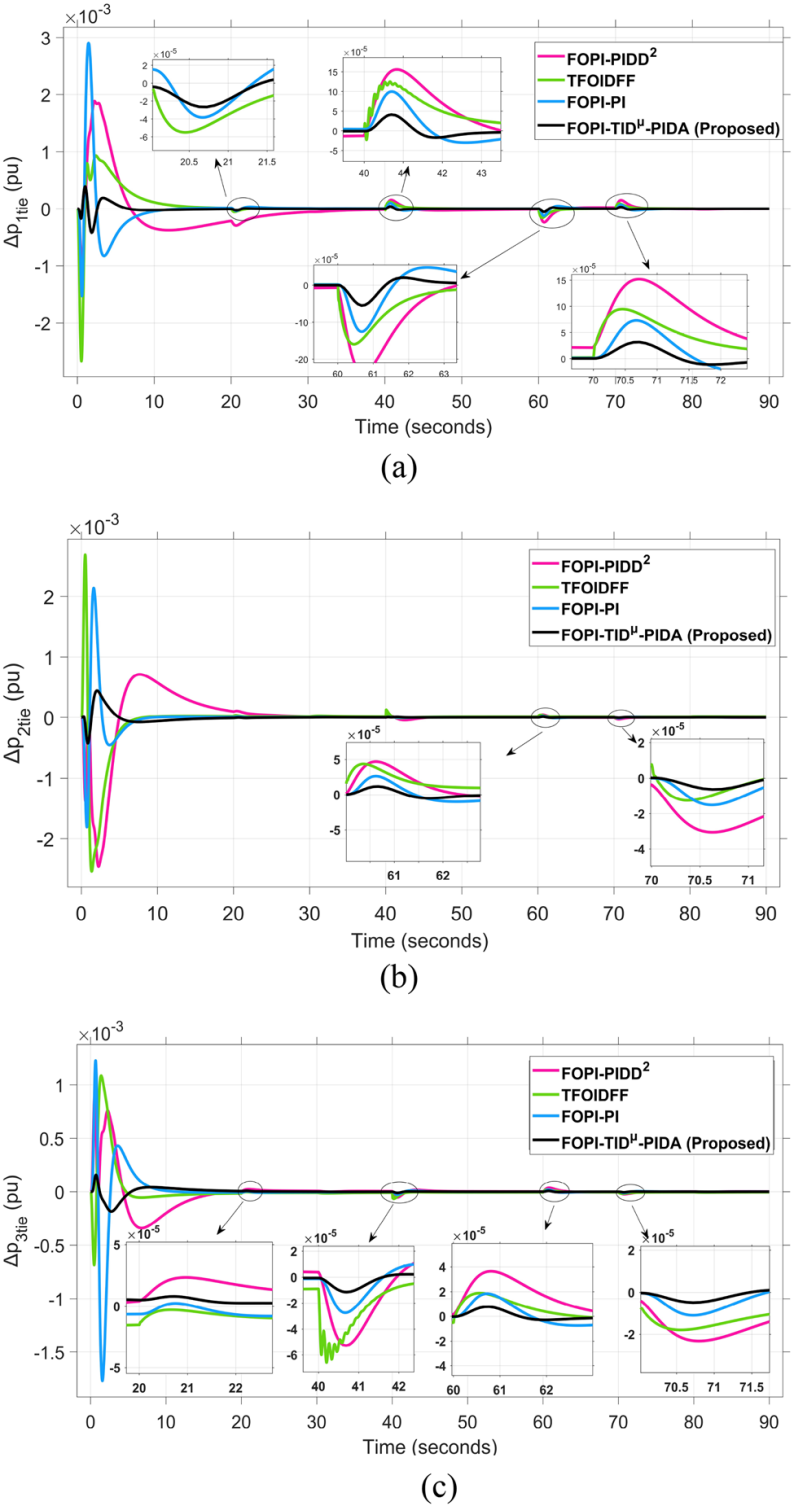
The fluctuation of tie-line power performance in Scenario 3 for the triple-area system when using different controllers tuned via DCS technique: (**a**) ∆P_1tie_, (**b**) ∆P_2tie_, and (**c**) ∆P_3tie_.

For all considered controllers based on the proposed DCS algorithm for tuning their parameters, the resultant profiles for voltage, frequency, and tie-line power for the three considered areas are shown in Figures 19, 20, and 21, respectively. From figure 19, for Area 1, the voltage response V₁, shows that the FOPI-PIDD^2^ controller yields the highest overshoot, peaking at approximately 1.182 pu during the transient response phase. From figure 20, in Area 3, the frequency deviation ΔF_3_ indicates that the FOPI-PI controller results in the deepest undershoot, approximately −0.014 Hz, compared to other competing control structures. In figure 21, for Area 2, the tie-line power deviation ΔP_tie2_ (Figure 20b) reveals that the TFOIDFF controller reaches a maximum overshoot of 1.182 pu, indicating its sensitivity to stochastic fluctuations within the interconnected network.

On the other hand, the proposed FOPI-TID^µ^-PIDA regulator demonstrates exceptional capability in both Load Frequency Control (LFC) and Automatic Voltage Regulation (AVR) loops. It achieves the lowest undershoot, overshoot, and settling time, effectively managing both rapid and gradual load variations. All obtained key performance metrics for all controllers in the three considered areas of the power system are summarized in Table 6.

**Table 6:** The dynamic performance of the studied system when using different DCS-based controllers under the effect of Scenario 3.

**Controller**	**Features**	**V**_**1(pu)**_	**V**_**2(pu)**_	**V**_**3(pu)**_	**ΔF**_**1(Hz)**_	**ΔF**_**2(Hz)**_	**ΔF**_**3(Hz)**_	**ΔP**_**tie1(pu)**_	**ΔP**_**tie2(pu)**_	**ΔP**_**tie3(pu)**_
**FOPI-PIDD**^**2**^	**POS**	1.182	1.126	1.08	0.028	0.03	0.031	1.8 x10^−3^	7.1 x10^−4^	9 x10^−4^
	**PUS**	0.822	0.928	0.864	−3.8 x10^−3^	−2.6 x10^−3^	−0.004	9.3 x10^−3^	−2.4 x10^−3^	−3.4 x10^−4^
	**Tst**	8	9	6	18	18	19	36	28	24
**TFOIDFF**	**POS**	1.076	1.15	1.091	0.023	0.029	0.024	−2.6 x10^−3^	2.6 x10^−3^	1 x10^−3^
	**PUS**	0.920	0.983	0.961	−8 x10^−3^	−3.1 x10^−3^	−0.001	−9 x10^−3^	−2.5 x10^−3^	−7 x10^−4^
	**Tst**	15	5	8	14	12	19	18	10	24
**FOPI-PI**	**POS**	1.133	1.120	1.06	0.018	0.015	0.023	0.003	2.1 x10^−3^	1.2 x10^−3^
	**PUS**	0.995	0.993	0.985	−8.5 x10^−3^	−7.2 x10^−3^	−0.014	−1.5 x10^−3^	−1.8 x10^−3^	−1.7 x10^−3^
	**Tst**	7	4	8	10	10	10	15	10	14
**FOPI-TID**^**µ**^**-PIDA** **(Proposed)**	**POS**	1.051	1.06	1.063	6.2 x10^−3^	4.3 x10^−3^	6 x10^−3^	3.9 x10^−4^	4 x10^−4^	1.5 x10^−4^
	**PUS**	0.970	0.957	0	−2 x10^−3^	−1.3 x10^−3^	−1.3 x10^−3^	−4 x10^−4^	−4 x10^−4^	−1.8 x10^−4^
	**Tst**	5	4	5	8	8	7	10	28	20

#### SCENARIO 4: Applying unpredictable multi-stage load in area 1 as well as making variation in voltage reference for the three difference areas

**Fig. 22:**
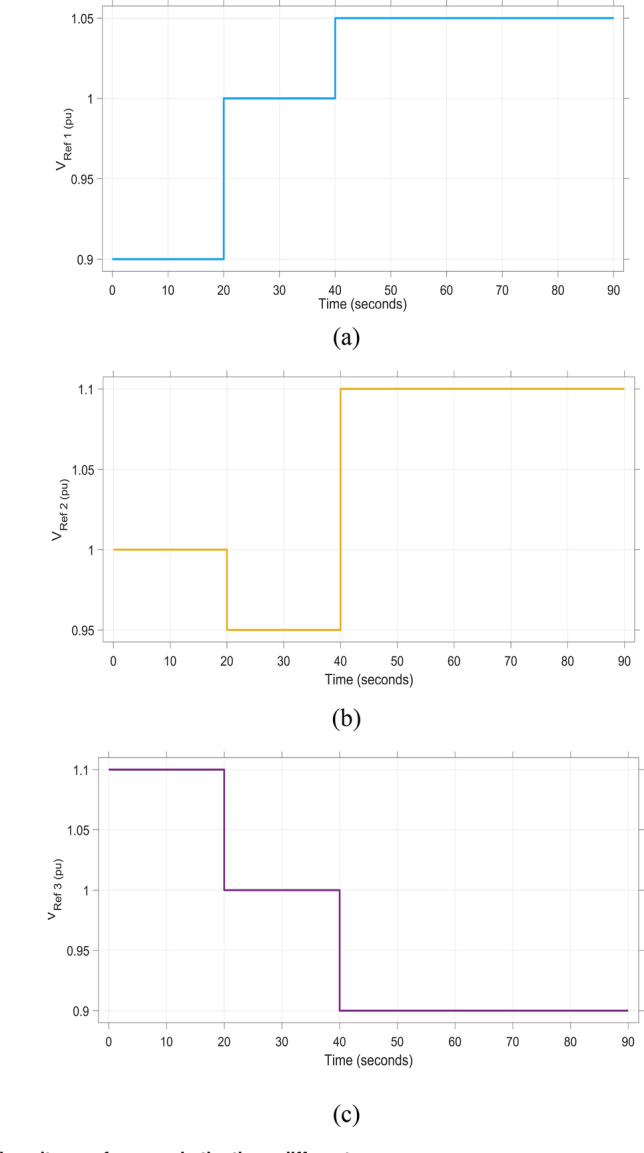
The variation profiles in voltage references in the three different areas.

**Fig. 23:**
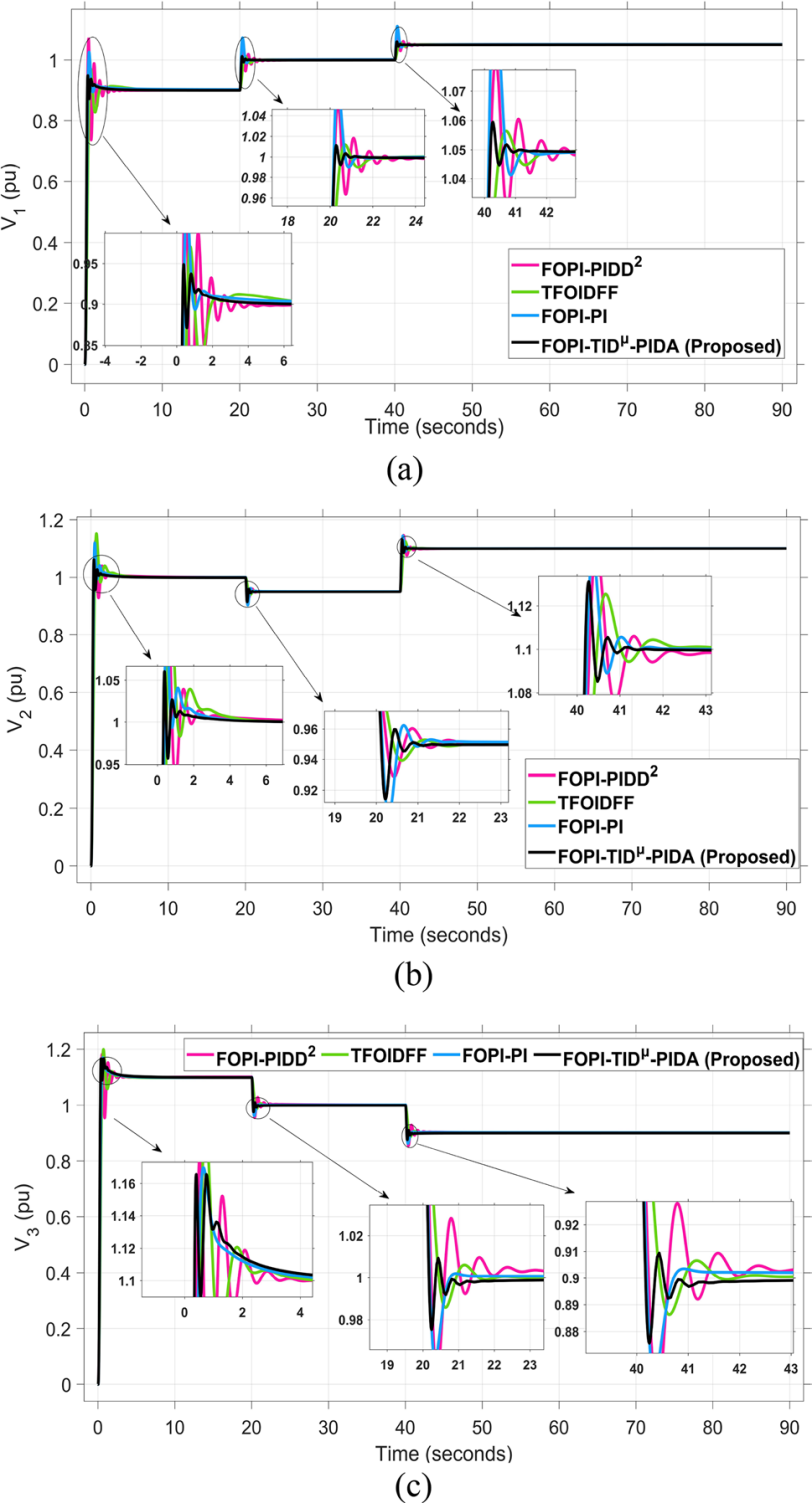
The voltage performance in Scenario 4 for the triple-area system when using different controllers tuned via DCS technique: (**a**) V_1_, (**b**) V_2_, and (**c**) V_3_.

**Fig. 24:**
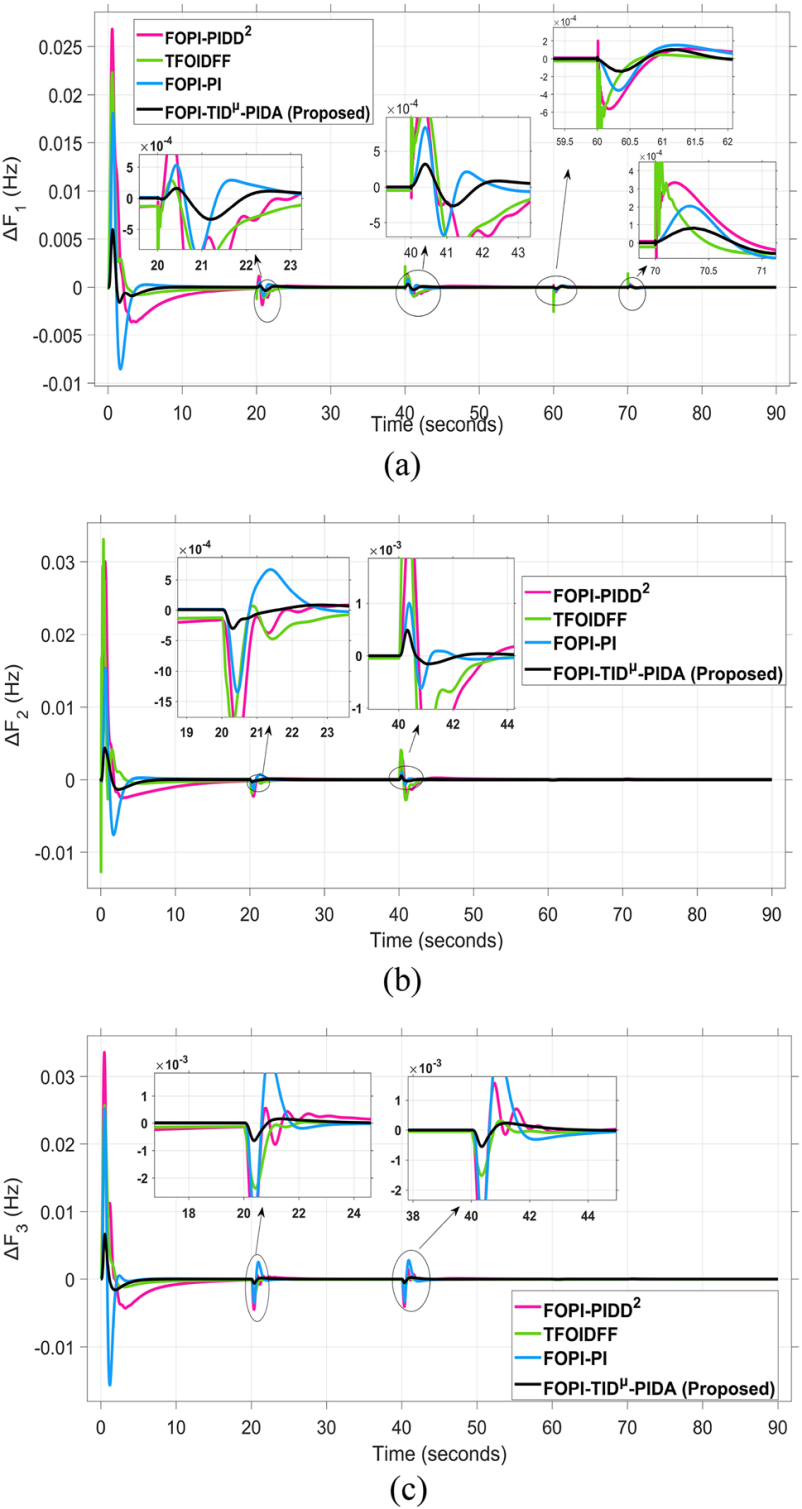
The effectiveness of frequency regulation in Scenario 4 for the triple-area model when using different controllers tuned via DCS technique: (**a**) ∆F_1_, (**b**) ∆F_2_, and (**c**) ∆F_3_.

**Fig. 25:**
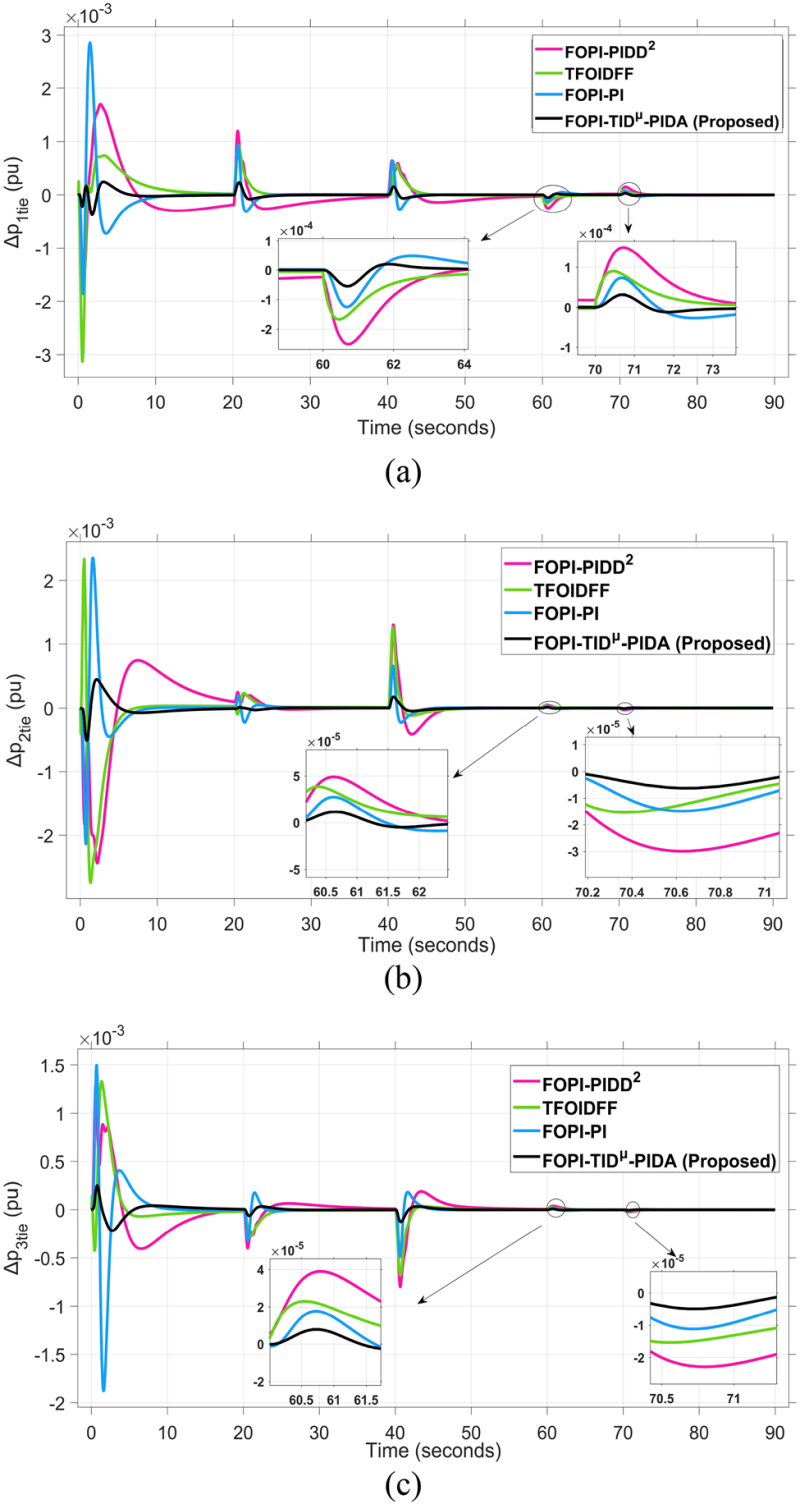
The fluctuation of tie-line power performance in Scenario 4 for the triple-area system when using different controllers tuned via DCS technique: (**a**) ∆P_1tie_, (**b**) ∆P_2tie_, and (**c**) ∆P_3tie_.

In this Scenario, an unpredictable multi-step load disturbance in Area 1 to investigate its impact on the performance of the proposed FOPI-TID^µ^-PIDA controller in enhancing the dynamic performance of a hybrid power system. The load profiles in the three considered areas are illustrated in Figure 22. Accordingly, the system`s performance parameters are depicted in Figures 23, 24, and 25. From Figure 23, in Area 1, the FOPI-PIDD^2^ controller exhibits the highest overshoot for the parameter V_1_ of approximately 1.066 pu during the transient phase. Moreover, under the combined influence of load fluctuation and time-varying voltage setpoints, the FOPI-PI controller produces the most pronounced peak response compared to the other schemes operating within the same region. From Figure 24, in Area 2, the frequency deviation response ΔF shows that the FOPI-PIDD^2^ controller maintains the longest settling time, reaching up to 18 seconds. Despite the fluctuations in reference voltage, this controller continues to exhibit delayed convergence toward SteadyState, highlighting its relatively slower dynamic behavior in this case. From Figure 25, for Area 3, the power deviation across the tie-line ΔP_tie3_ shows that the FOPI-PI controller induces the maximum undershoot, reaching approximately −1.8 × 10⁻^3^ pu, among all evaluated controllers.

In contrast, the proposed FOPI-TID^µ^-PIDA controller rapidly dampens oscillations, showcasing the lowest undershoot and overshoot. Therefore, the optimized parameters by the proposed DCS algorithm for the proposed controller maintain superior control quality when handling both sudden and gradual load variations under time-varying reference voltage conditions. All numerical values for the system`s parameters for the considered controllers for this scenario are summarized in Table 7.

**Table 7:** The dynamic performance of the studied system when using different DCS-based controllers under the effect of Scenario 4.

**Controller**	**Features**	**V**_**1(pu)**_	**V**_**2(pu)**_	**V**_**3(pu)**_	**ΔF**_**1(Hz)**_	**ΔF**_**2(Hz)**_	**ΔF**_**3(Hz)**_	**ΔP**_**tie1(pu)**_	**ΔP**_**tie2(pu)**_	**ΔP**_**tie3(pu)**_
**FOPI-PIDD**^**2**^	**POS**	1.066	1.126	1.8	0.026	0.03	0.033	1.7 x10^−3^	7 x10^−4^	1.2 x10^−3^
	**PUS**	−0.737	−0.928	−0.954	−0.003	−0.002	−0.004	−1.7 x10^−3^	−2.4 x10^−3^	−4 x10^−4^
	**Tst**	7	9	5	18	18	18	40	30	20
**TFOIDFF**	**POS**	0.969	1.151	1.2	0.022	0.033	0.026	7.4 x10^−4^	2.4 x10^−3^	1.3 x10^−3^
	**PUS**	−0.827	−0.983	−1.058	−8 x10^−4^	−0.012	1.1 x10^−3^	−0.003	−2.7 x10^−3^	−4.2 x10^−4^
	**Tst**	8	5.5	9	16	12	10	18	14	16
**FOPI-PI**	**POS**	1.025	1.12	1.17	0.018	0.015	0.025	0.003	2.3 x10^−3^	1.5 x10^−3^
	**PUS**	−0.893	−0.993	0	−0.008	−0.007	−0.015	−0.002	−2.1 x10^−3^	−1.8 x10^−3^
	**Tst**	8	4	6	10	10	8	16	10	12
**FOPI-TID**^**µ**^**-PIDA** **(Proposed)**	**POS**	0.948	1.06	1.165	0.006	0.004	0.006	2.4 x10^−4^	4 x10^−4^	2 x10^−4^
	**PUS**	−0.871	−0.957	−1.089	−0.001	−0.001	−1.5 x10^−3^	−3.7 x10^−4^	−5 x10^−4^	−2 x10^−4^
	**Tst**	6	4	7	10	10	7	8	16	18

#### SCENARIO 5: Conducting a sensitivity analysis to assess the robustness of the controllers under different operating conditions

This section evaluates the robustness of the proposed FOPI-TIDµ-PIDA controller by varying the system parameters within a defined range of $\pm 25\boldsymbol{\%}$, including ${\boldsymbol{B}}$, ${\boldsymbol{G}}{\boldsymbol{B}}$, ${{\boldsymbol{K}}}_{{\boldsymbol{w}}1}$, ${{\boldsymbol{K}}}_{{\boldsymbol{w}}2}$, ${{\boldsymbol{K}}}_{{\boldsymbol{d}}{\boldsymbol{i}}}$, ${\boldsymbol{R}}$, ${{\boldsymbol{T}}}_{12}$, ${{\boldsymbol{T}}}_{13}$, ${{\boldsymbol{T}}}_{23}$, ${{\boldsymbol{T}}}_{{\boldsymbol{R}}}$, ${{\boldsymbol{T}}}_{{\boldsymbol{w}}1}$, ${{\boldsymbol{T}}}_{{\boldsymbol{w}}2}$, ${\boldsymbol{A}}12$, ${\boldsymbol{A}}13$, ${\boldsymbol{A}}23$, ${{\boldsymbol{b}}}_{{\boldsymbol{p}}{\boldsymbol{v}}}$, and ${{\boldsymbol{c}}}_{{\boldsymbol{p}}{\boldsymbol{v}}}$. The sensitivity analysis is conducted to verify the controller’s capability to preserve system stability under parameter uncertainties without altering the optimized settings of the proposed FOPI-TIDµ-PIDA controller. The evaluation was performed under the same conditions as Scenario 1, incorporating a 10% step load disturbance (SLD) in Area 1. The analysis indicates that no impact on the voltage response across the three areas, as it remained consistent with the nominal value. However, there are minor impacts on both ${\Delta {\boldsymbol{F}}}_{{\boldsymbol{i}}}$ and ${\Delta {\boldsymbol{P}}}_{{\boldsymbol{i}}{\boldsymbol{j}}}$ due to the parameter variations. The peak overshoot (POS) and peak undershoot (PUS) displayed minimal deviations compared to normal operating conditions. These results confirm that the proposed controller is robust and reliable, effectively maintaining voltage stability, managing frequency deviations, and regulating tie-line power flow under diverse operating scenarios. The effect of the proposed controller`s parameters changing on ${\Delta {\boldsymbol{F}}}_{{\boldsymbol{i}}}$ and ${\Delta {\boldsymbol{P}}}_{{\boldsymbol{i}}{\boldsymbol{j}}}$ are summarized in Table 8 and Table 9, respectively.

**Table 8:** The system dynamic characteristics of ∆F under parametric variations.

**Parameter**	**%Change**	**ΔF**_**1 (Hz)**_			**ΔF**_**2 (Hz)**_			**ΔF**_**3 (Hz)**_		
		**POS**	**PUS**	**Tst**	**POS**	**PUS**	**Tst**	**POS**	**PUS**	**Tst**
**Normal**	$0$	0.006	−0.001	6.483	0.004	−0.001	4	0.006	−0.001	4
${\boldsymbol{B}}$	$+25\%$	0.005	−0.001	6.593	0.004	−0.001	4.09	0.004	−0.001	3.82
	$-25\%$	0.008	−0.002	6.403	0.004	−0.001	3.93	0.008	−0.001	4.17
${\boldsymbol{G}}{\boldsymbol{B}}$	$+25\%$	0.006	−0.001	6.493	0.004	−0.001	4.02	0.006	−0.001	3.99
	$-25\%$	0.006	−0.001	6.483	0.004	−0.001	3.99	0.006	−0.001	4
${{\boldsymbol{K}}}_{{\boldsymbol{w}}1}$	$+25\%$	0.006	−0.001	6.493	0.004	−0.001	4.02	0.006	−0.001	3.99
	$-25\%$	0.006	−0.001	6.483	0.004	−0.001	3.99	0.006	−0.001	4
${{\boldsymbol{K}}}_{{\boldsymbol{w}}2}$	$+25\%$	0.006	−0.001	6.493	0.004	−0.001	4.02	0.006	−0.001	3.99
	$-25\%$	0.006	−0.001	6.483	0.004	−0.001	3.99	0.006	−0.001	4
${{\boldsymbol{K}}}_{{\boldsymbol{d}}{\boldsymbol{i}}}$	$+25\%$	0.005	−0.001	6.453	0.004	−0.001	3.99	0.004	−0.001	4.01
	$-25\%$	0.008	−0.002	6.533	0.004	−0.001	4.02	0.008	−0.001	3.97
${\boldsymbol{R}}$	$+25\%$	0.006	−0.001	6.473	0.004	−0.001	3.99	0.006	−0.001	3.99
	$-25\%$	0.006	−0.001	6.503	0.004	−0.001	4.03	0.006	−0.001	4.02
${{\boldsymbol{T}}}_{12}$	$+25\%$	0.006	−0.001	6.483	0.004	−0.001	4.01	0.006	−0.001	4
	$-25\%$	0.006	−0.002	6.483	0.004	−0.001	4	0.006	−0.001	4
${{\boldsymbol{T}}}_{13}$	$+25\%$	0.006	−0.001	6.413	0.004	−0.001	3.99	0.006	−0.001	4.04
	$-25\%$	0.006	−0.001	6.593	0.004	−0.001	4.02	0.006	−0.001	3.94
${{\boldsymbol{T}}}_{23}$	$+25\%$	0.006	−0.001	6.463	0.004	−0.001	3.93	0.006	−0.001	4.08
	$-25\%$	0.006	−0.001	6.523	0.004	−0.001	4.12	0.006	−0.001	3.85
${{\boldsymbol{T}}}_{{\boldsymbol{R}}}$	$+25\%$	0.006	−0.001	6.493	0.004	−0.001	4.01	0.006	−0.001	4.01
	$-25\%$	0.006	−0.001	6.477	0.004	−0.001	3.994	0.006	−0.001	3.994
${{\boldsymbol{T}}}_{{\boldsymbol{w}}1}$	$+25\%$	0.006	−0.001	6.481	0.004	−0.001	3.998	0.006	−0.001	3.998
	$-25\%$	0.006	−0.001	6.483	0.004	−0.001	4	0.006	−0.001	4
${{\boldsymbol{T}}}_{{\boldsymbol{w}}2}$	$+25\%$	0.006	−0.001	6.463	0.004	−0.001	3.98	0.006	−0.001	3.97
	$-25\%$	0.006	−0.001	6.513	0.004	−0.001	4.03	0.006	−0.001	4.03
${\boldsymbol{A}}12$	$+25\%$	0.006	−0.001	6.493	0.004	−0.001	4.02	0.006	−0.001	4.03
	$-25\%$	0.006	−0.001	6.483	0.004	−0.001	3.99	0.006	−0.001	3.98
${\boldsymbol{A}}13$	$+25\%$	0.006	−0.001	6.543	0.004	−0.001	4.09	0.006	−0.001	4.12
	$-25\%$	0.006	−0.001	6.433	0.004	−0.001	3.92	0.006	−0.001	3.89
${\boldsymbol{A}}23$	$+25\%$	0.006	−0.001	6.523	0.004	−0.001	4	0.006	−0.001	3.98
	$-25\%$	0.006	−0.002	6.443	0.004	−0.001	4	0.006	−0.001	4.02
${{\boldsymbol{b}}}_{{\boldsymbol{p}}{\boldsymbol{v}}}$	$+25\%$	0.006	−0.002	6.453	0.004	−0.001	3.98	0.006	−0.001	3.99
	$-25\%$	0.006	−0.001	6.523	0.004	−0.001	4.03	0.006	−0.001	4.03
${{\boldsymbol{c}}}_{{\boldsymbol{p}}{\boldsymbol{v}}}$	$+25\%$	0.006	−0.001	6.483	0.004	−0.001	4.02	0.006	−0.001	4.03
	$-25\%$	0.006	−0.001	6.483	0.004	−0.001	3.97	0.006	−0.001	3.96

**Table 9:** The system dynamic characteristics of ∆P_tie_ under parametric variation.

**Parameter**	**%Change**	**ΔP**_**1tie (pu)**_			**ΔP**_**2tie (pu)**_			**ΔP**_**3tie (pu)**_		
		**POS**	**PUS**	**Tst**	**POS**	**PUS**	**Tst**	**POS**	**PUS**	**Tst**
**Normal**	$0$	0.0003	−0.0004	6	0.0001	−0.0004	7.5	0.0001	−0.0001	12
${\boldsymbol{B}}$	$+25\%$	0.0003	−0.0004	6.72	0.0001	−0.0003	7.677	0.0001	−0.0001	12.12
	$-25\%$	0.0003	0.0004	5.3	0.0001	−0.0004	6.96	0.0001	−0.0001	12
${\boldsymbol{G}}{\boldsymbol{B}}$	$+25\%$	0.0003	0.0004	5.96	0.0001	−0.0004	7.5	0.0001	−0.0001	12
	$-25\%$	0.0003	0.0004	6.04	0.0001	−0.0004	7.51	0.0001	−0.0001	12.23
${{\boldsymbol{K}}}_{{\boldsymbol{w}}1}$	$+25\%$	0.0003	0.0004	6.01	0.0001	−0.0004	7.5	0.0001	−0.0001	12
	$-25\%$	0.0003	0.0004	6	0.0001	−0.0004	7.5	0.0001	−0.0001	12
${{\boldsymbol{K}}}_{{\boldsymbol{w}}2}$	$+25\%$	0.0003	0.0004	5.96	0.0001	−0.0004	7.5	0.0001	−0.0001	12.13
	$-25\%$	0.0003	0.0004	6.04	0.0001	−0.0004	7.51	0.0001	−0.0001	12.05
${{\boldsymbol{K}}}_{{\boldsymbol{d}}{\boldsymbol{i}}}$	$+25\%$	0.0003	0.0003	5.96	0.0001	−0.0004	7.5	0.0001	−0.0001	12
	$-25\%$	0.0003	0.0005	6.04	0.0001	−0.0004	7.51	0.0001	−0.0001	12
${\boldsymbol{R}}$	$+25\%$	0.0003	0.0004	5.45	0.0001	−0.0004	7.49	0.0001	−0.0001	11.98
	$-25\%$	0.0003	0.0004	6.38	0.0001	−0.0004	7.51	0.0001	−0.0001	12.03
${{\boldsymbol{T}}}_{12}$	$+25\%$	0.0003	0.0004	5.94	0.0001	−0.0004	7.46	0.0001	−0.0001	11.739
	$-25\%$	0.0003	0.0004	6.1	0.0001	−0.0004	7.57	0.0001	−0.0001	12.325
${{\boldsymbol{T}}}_{13}$	$+25\%$	0.0003	0.0004	5.57	0.0001	−0.0004	7.38	0.0001	−0.0001	11.114
	$-25\%$	0.0003	0.0004	6.59	0.0001	−0.0004	7.65	0.0001	−0.0001	12.122
${{\boldsymbol{T}}}_{23}$	$+25\%$	0.0003	0.0004	5.71	0.0001	−0.0004	7.5	0.0001	−0.0001	12
	$-25\%$	0.0003	0.0004	6.36	0.0001	−0.0004	7.5	0.0001	−0.0001	12
${{\boldsymbol{T}}}_{{\boldsymbol{R}}}$	$+25\%$	0.0003	0.0004	6.04	0.0001	−0.0004	7.15	0.0001	−0.0001	11.69
	$-25\%$	0.0003	0.0004	5.97	0.0001	−0.0004	7.677	0.0001	−0.0001	12.25
${{\boldsymbol{T}}}_{{\boldsymbol{w}}1}$	$+25\%$	0.0003	0.0004	5.99	0.0001	−0.0004	7.5	0.0001	−0.0001	12.13
	$-25\%$	0.0003	0.0004	6.01	0.0001	−0.0004	7.5	0.0001	−0.0001	12
${{\boldsymbol{T}}}_{{\boldsymbol{w}}2}$	$+25\%$	0.0003	0.0004	5.99	0.0001	−0.0004	7.51	0.0001	−0.0001	12
	$-25\%$	0.0003	0.0004	6.01	0.0001	−0.0004	7.49	0.0001	−0.0001	12
${\boldsymbol{A}}12$	$+25\%$	0.0003	0.0004	6	0.0001	−0.0004	7.5	0.0001	−0.0001	12
	$-25\%$	0.0003	0.0004	6	0.0001	−0.0004	7.51	0.0001	−0.0001	12
${\boldsymbol{A}}13$	$+25\%$	0.0003	0.0004	6.01	0.0001	−0.0004	7.49	0.0001	−0.0001	12
	$-25\%$	0.0003	0.0004	5.99	0.0001	−0.0004	7.51	0.0001	−0.0001	12.06
${\boldsymbol{A}}23$	$+25\%$	0.0003	0.0004	5.98	0.0001	−0.0004	7.46	0.0001	−0.0001	11.93
	$-25\%$	0.0003	0.0004	6.024	0.0001	−0.0004	7.544	0.0001	−0.0001	12.07
${{\boldsymbol{b}}}_{{\boldsymbol{p}}{\boldsymbol{v}}}$	$+25\%$	0.0003	0.0004	5.998	0.0001	−0.0004	7.508	0.0001	−0.0001	12
	$-25\%$	0.0003	0.0004	6	0.0001	−0.0004	7.5	0.0001	−0.0001	12
${{\boldsymbol{c}}}_{{\boldsymbol{p}}{\boldsymbol{v}}}$	$+25\%$	0.0003	0.0004	6.2	0.0001	−0.0004	7.3	0.0001	−0.0001	11.76
	$-25\%$	0.0003	0.0004	5.79	0.0001	−0.0004	7.677	0.0001	−0.0001	12.11

#### SCENARIO 6: Evaluating controller resilience under cyber-attack conditions in smart grid environments

**Fig. 26:**
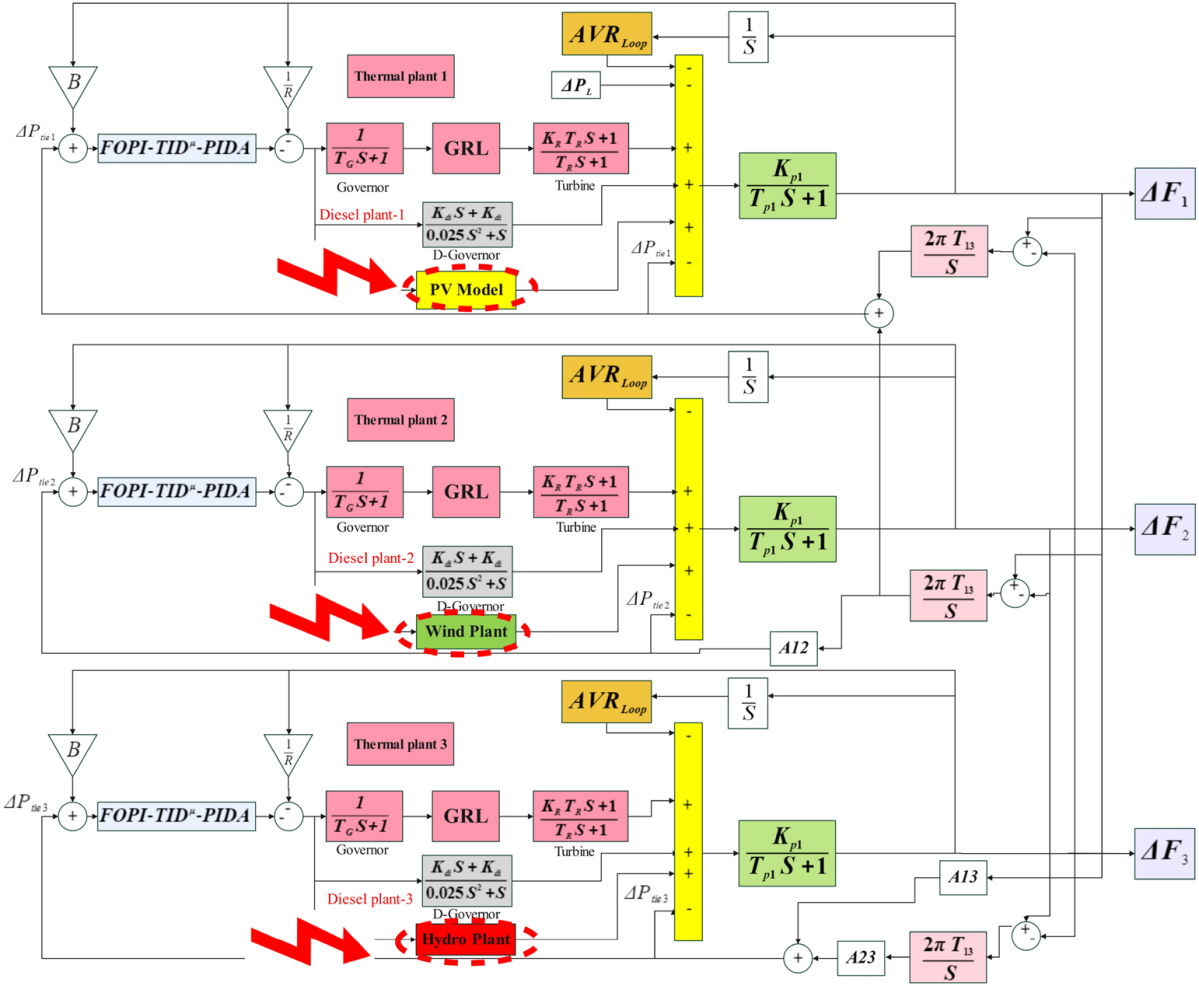
Considered Scenarios of Denial-of-Service Cyber Attack for the system under study.

To assess the controller’s performance during cyber-attacks in smart grid environment, a Denial-of-Service (DoS) scenario was simulated, targeting renewable energy units in three interconnected hybrid regions, as seen in Figure 26. As mentioned before, each region contains a different renewable source: PV in Area 1, wind in Area 2, and hydro in Area 3. The study examined four scenarios: (1) DoS on solar in Area 1, (2) DoS on wind in Area 2, (3) DoS on hydro in Area 3, and (4) a combined DoS attack on all renewables simultaneously. In each case, the affected units were taken offline while others stayed active. During the simulation of this scenario, Voltage responses were also checked but showed minimal changes, so they were excluded for simplicity. Therefore, the system’s reaction was analyzed based on tie-line power fluctuations ${\Delta {\boldsymbol{F}}}_{{\boldsymbol{i}}}$ and frequency variations ${\Delta {\boldsymbol{P}}}_{{\boldsymbol{i}}{\boldsymbol{j}}}$.

**Fig. 27:**
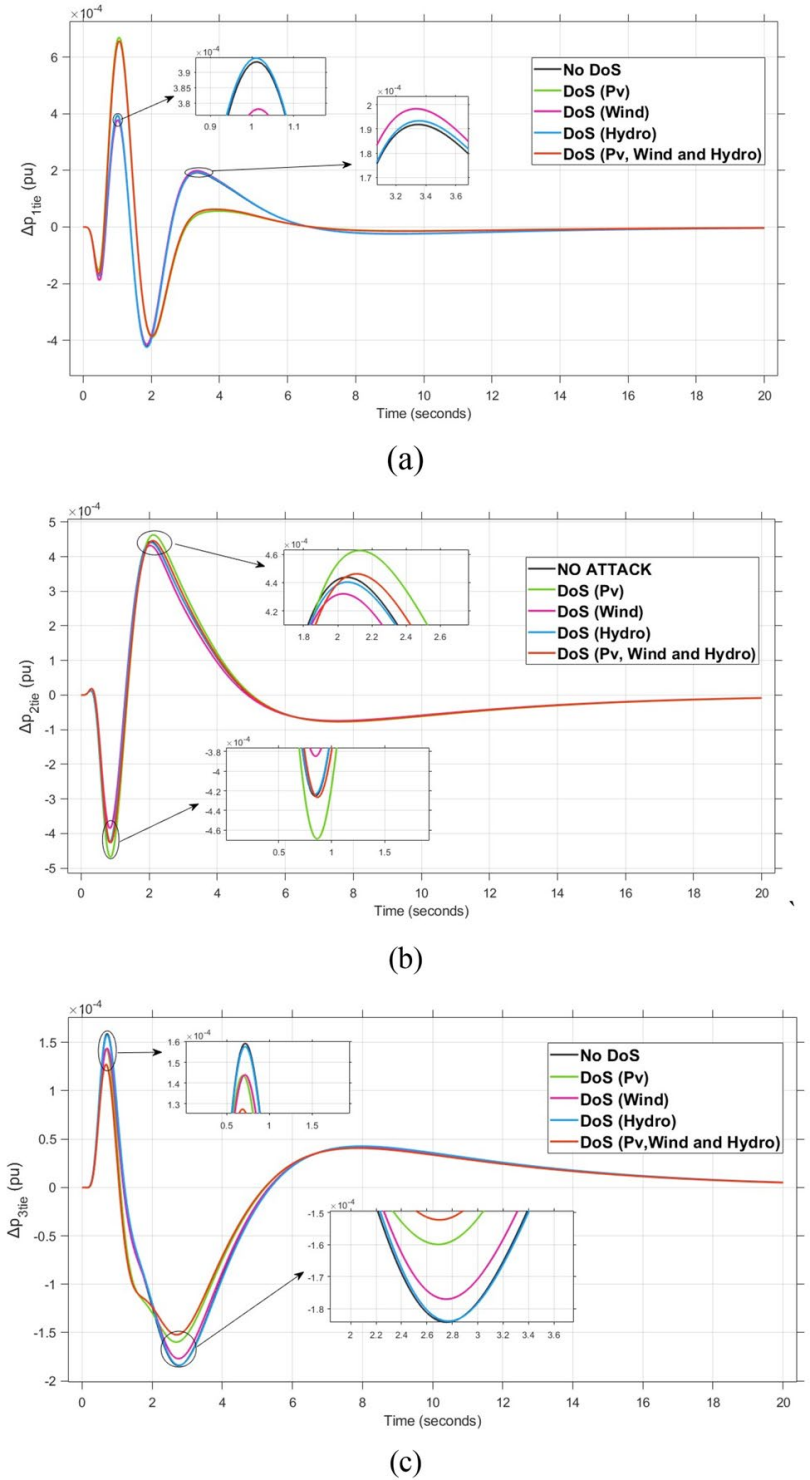
The fluctuation of tie-line power performance in Scenario 6 for the triple-area system during applying Dos attack on different RES units with proposed controllers tuned via DCS technique: (**a**) ∆P_1tie_, (**b**) ∆P_2tie_, and (**c**) ∆P_3tie_.

**Fig. 28:**
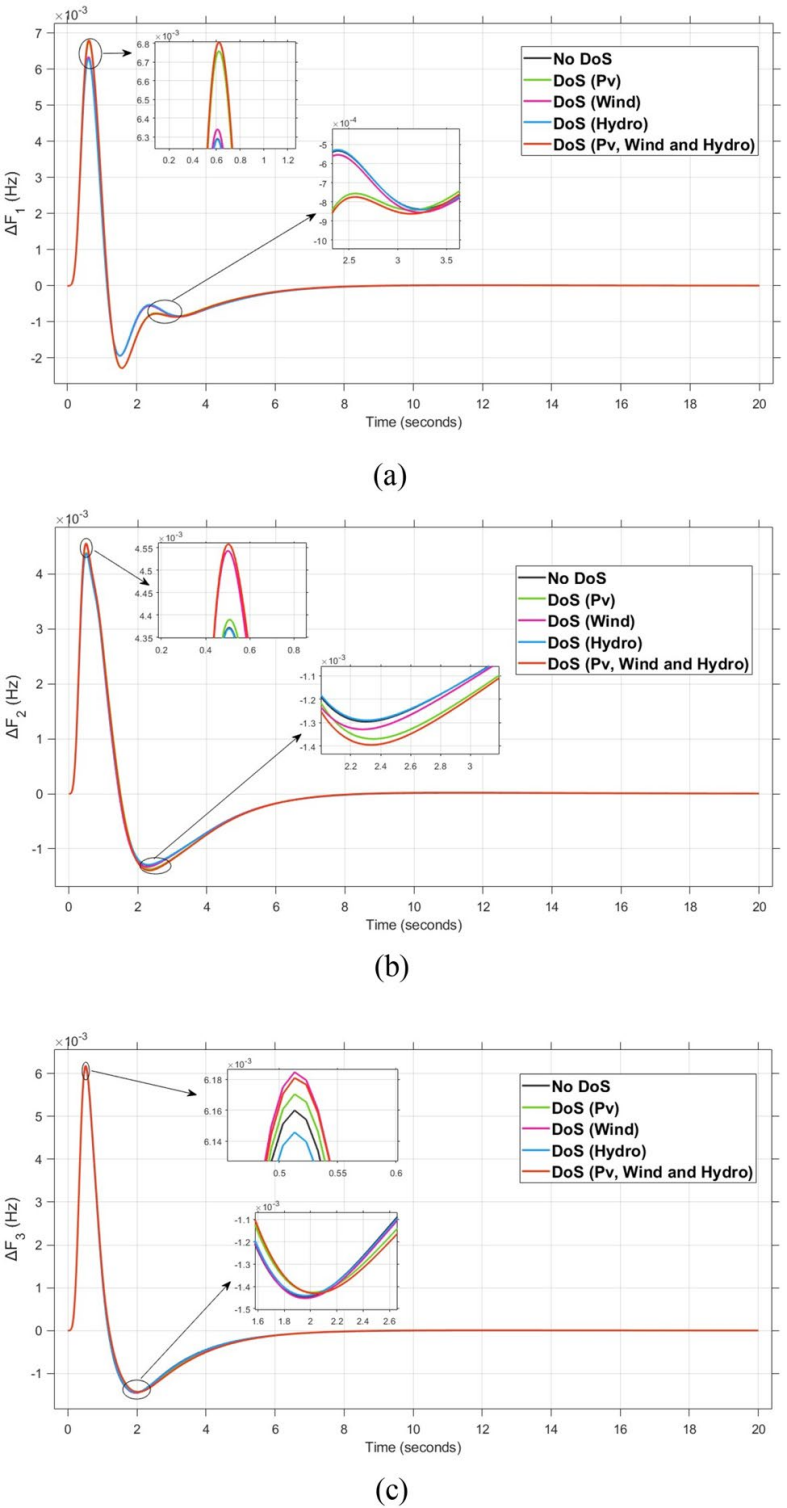
The effectiveness of frequency regulation in Scenario 6 for the triple-area model system during applying Dos attack on different RES units with proposed controllers tuned via DCS technique: (**a**) ∆F_1_, (**b**) ∆F_2_, and (**c**) ∆F_3_.

Power and frequency deviations are illustrated in Figures 27 and 28, respectively. From the figure, the displayed patterns depend on the attack scenario. In Area 1 (${\boldsymbol{\Delta }{\boldsymbol{P}}}_{1\boldsymbol{ }{\boldsymbol{t}}{\boldsymbol{i}}{\boldsymbol{e}}}$), cutting off solar leads to faster damping of the second overshoot compared to normal operation, while wind and hydro disruptions caused smaller adjustments. However, the full DoS attack produces a stronger disturbance, the peak is around 6.5×10⁻^4^ p.u. before stabilizing.

In Area 3 (${\boldsymbol{\Delta }{\boldsymbol{P}}}_{3\boldsymbol{ }{\boldsymbol{t}}{\boldsymbol{i}}{\boldsymbol{e}}}$), the hydro loss had a major impact, generating larger overshoots and deeper dips than individual solar or wind outages. In Figure 28d, the simultaneous attack results in the worst performance decline. Frequency deviation results in Area 3 (${\boldsymbol{\Delta }{\boldsymbol{F}}}_{3}$) shows that all attacks increased the initial overshoot. The combined DoS scenario reached the highest peak at roughly 6.2×10⁻^3^ Hz, compared to 6.14×10⁻^3^ Hz under normal conditions.

Therefore, among single-unit attacks, hydro disconnection had the most severe effect, highlighting its role in maintaining system inertia. Additionally, the undershoot after the initial peak worsened in all attack cases, with the deepest drop occurring during the full DoS event.

To sum up, while the system remains stable in all scenarios, the power and frequency responses indicates that losing renewable inputs especially during widespread DoS attacks degrades performance. The controller manages this to keep settling times within acceptable limits, but the larger oscillations emphasize the need for robust cybersecurity measures and resilient renewable integration in power grids.

### Descation between the different dcs-based controllers

**Fig. 29:**
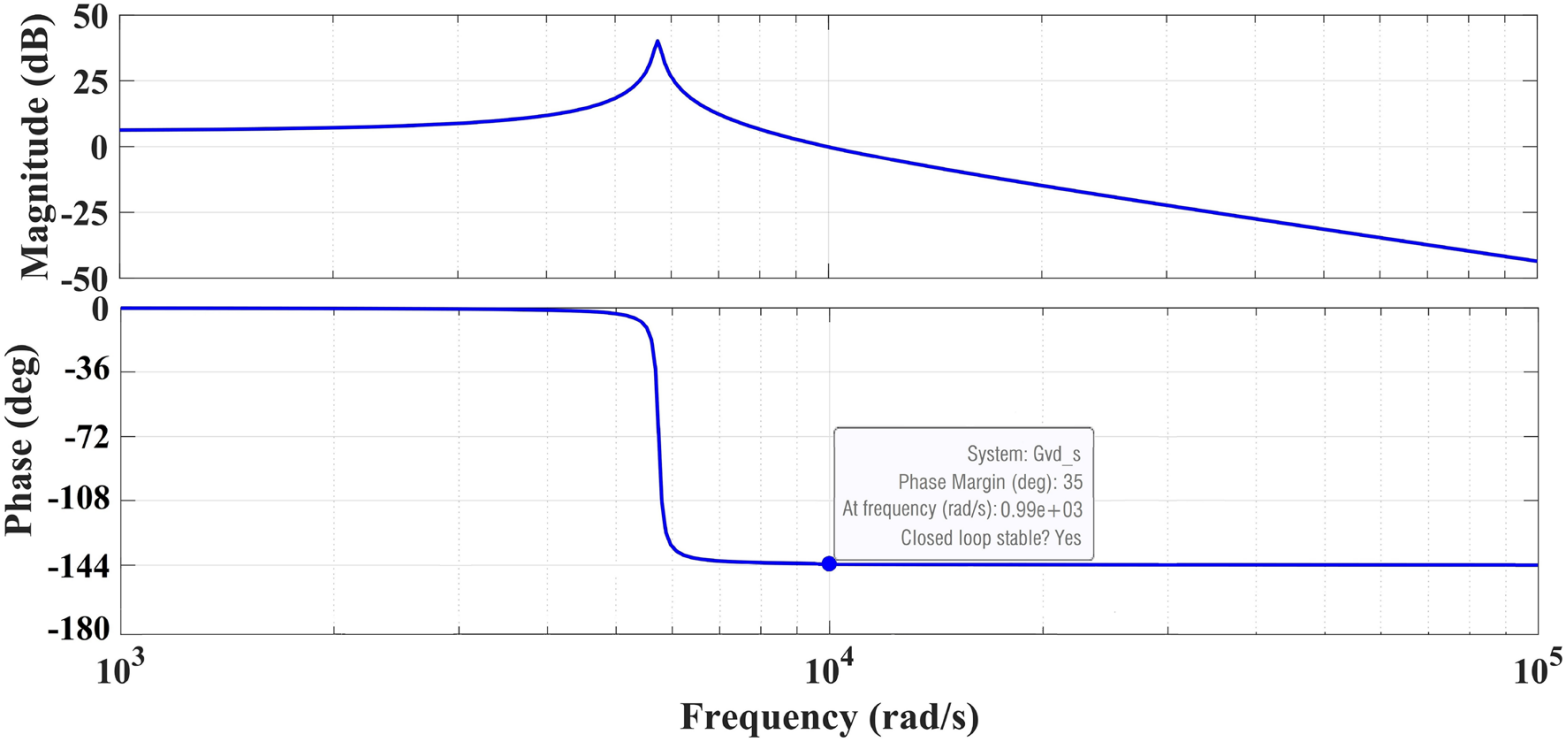
Bode plot shows the stability analysis of the proposed system.

In this study, the settling time (Ts) was calculated as the time required for the system response to remain within a predefined tolerance band of ±2% around the final steady-state value. This criterion was consistently applied to the frequency deviation (${\boldsymbol{\Delta }{\boldsymbol{F}}}_{{\boldsymbol{x}}}$), tie-line power (${\boldsymbol{\Delta }{\boldsymbol{P}}}_{{\boldsymbol{x}}{\boldsymbol{y}}}$), and terminal voltage response across all three control areas. The selection of the ±2% band is in line with standard control engineering practice and allows for a fair comparison of transient performance among the different controllers under identical load disturbance scenarios. The FOPI-TIDμ-PIDA cascade controller was selected over advanced schemes like MPC and ANFIS due to its superior adaptability, simplicity, and implementation efficiency in complex power systems. Additionally, stability analysis was conducted to show the stability of the system using the proposed controller as illustrated in Figure 29. Bode plot is shows a positive value of phase margin equal 35 that indicates a good stable condition.

Moreover, unlike MPC, which requires precise real-time models, or ANFIS, which depends on extensive training data, the proposed structure leverages fractional and tilt-integral dynamics to handle nonlinearities, time delays, and multi-source variability effectively. Its cascade form enables fine-tuned response across voltage and frequency loops without demanding high computational resources or model retraining make it ideal for hybrid interconnected grids under dynamic conditions. The adoption of the FOPI–TIDμ–PIDA cascaded structure was a strategic control design grounded in both theoretical principles and recent literature. Each component contributes unique advantages: FOPI ensures robust baseline control with tunable flexibility, TIDμ enhances transient response by managing delays and inertial mismatches, and PIDA adds anticipatory action for fast dynamic correction. This layered configuration offers a coordinated multi-timescale response essential for modern renewable-integrated, multi-area systems. Rather than evaluating each controller in isolation, integrated tuning was applied to maximize synergy under nonlinear and uncertain scenarios. Similar cascaded strategies have shown superior results in hybrid systems.

## Conclusions

Voltage and frequency stabilization in linked power networks (LPNs) remains a significant challenge due to nonlinear dynamics and fluctuating load patterns, particularly in hybrid generation environments incorporating thermal, diesel, photovoltaic, hydro, and wind sources. In this work, a novel FOPI–TIDµ–PIDA cascaded controller optimized using the Differential Creative Search (DCS) algorithm was proposed for coordinated Load Frequency Control (LFC) and Automatic Voltage Regulation (AVR) in a three-area interconnected system. To assess the effectiveness of the proposed design, an extensive comparative study was conducted.

- Stability enhancement improved by:
  - 28.187% over Artificial Ecosystem-based Optimization (AEO)
  - 35.741% over Dandelion Optimizer (DO)
  - 21.879% over Runge–Kutta Optimization (RUN)
- Exhibited the lowest Integral Absolute Error (IAE), the proposed controller has 0.0507 compared with:
  - 0689 for FOPI–PIDD2.
  - 0713 for TFOIDFF.
  - 0.1124 for FOPI–PI.
- Demonstrated superior dynamic performance across six scenarios, showing:
  - Reduced overshoot and undershoot
  - Faster settling time
  - Strong resilience against cyber-attack disturbances
- Maintained robust operation under ±25% parameter variations, confirming controller adaptability and stability margins.

Despite the promising performance of the proposed DCS-optimized FOPI–TIDµ–PIDA controller, several limitations remain. Validation relied solely on simulation environments, excluding real-world considerations such as sensor inaccuracies, actuator delays, and more advanced cybersecurity threats beyond denial-of-service (DoS) attacks. Additionally, the system model was constrained to a simplified three-area interconnected structure, which may limit generalizability to larger or more complex networks. The intricate controller architecture may also impose computational challenges for real-time implementation. Finally, the adoption of a single-objective optimization approach restricted exploration of trade-offs among competing performance metrics.

Although the results are encouraging, several limitations should be acknowledged. The evaluation relied solely on simulation and did not incorporate practical factors such as sensor imperfections, actuator latency, or more diverse cyberattacks beyond denial-of-service (DoS). Additionally, the study employed a three-area system model with simplified representations of thermal and renewable sources, which may limit the scalability of the system. The proposed controller’s complex architecture could also present computational challenges for real-time implementation, and the use of a single-objective optimization approach restricted the exploration of performance trade-offs.

Therefore, our current study opens the door for several prospective research directions. These include real-time hardware implementation, the integration of communication delay models, and deeper investigations into the controller’s cybersecurity robustness. Also, future works may include generator`s nonlinearities and constraints, coordination with energy storage systems (ESS), and experimental validation on microgrid. Furthermore, the AGC examined in this article may be evaluated using different recently published smart controllers and optimization methodologies. The outcomes of these tests can then be compared to the conclusions presented in this paper.

## Data Availability

"The data presented in this study are available in body of this paper"

## Appendix

**Table:**

Parameter of The Studied Triple Area System						
Generating Areas	Thermal System	Wind System	Diesel System	PV Plant	Hydro System	AVR Loop
$T=100 s$	${T}_{G}=0.08 s$	${T}_{w1}=0.6 s$	${K}_{di}=16.5$	${a}_{pv}=900$	${T}_{RH}=48.7s$	${K}_{A}=10$
$A12=-0.5$	${T}_{T}=0.3 s$	${T}_{w2}=0.041 s$		${b}_{pv}=-18$	${T}_{r}=5s$	${T}_{A}=0.1 s$
$A13=-0.25$	${T}_{R}=10 s$	${K}_{w1}=1.25$		${c}_{pv}=100$	${T}_{GH}=0.513 s$	${K}_{E}= 1$
$A23=-0.5$	${K}_{R}=0.5$	${K}_{w2}=1.4$		${d}_{pv}=50$	${T}_{W}=1 s$	${T}_{E}= 0.4 s$
${K}_{P1}=120$		$GB=1$			${T}_{ij}=0.08 s$	${K}_{f}= 0.8$
${T}_{P1}=20 s$					$R=2.4$	${T}_{f}= 1.4 s$
					$B=0.425$	${K}_{1}=1$
						$${K}_{2}= -0.1$$
						$${K}_{3}= 0.5$$
						$${K}_{4}= 1.4$$
						$${P}_{s}= 0.145$$
						$${K}_{S}=1$$
						$${T}_{S}= 0.05 s$$

## Abbreviations

**LPNs**: Linked power networks
**AGC**: Automatic generation control
**DCS**: Differential creative search
**DCS-FOPI-TID**^**μ**^**-PIDA**: Proposed cascaded controller tuned via DCS
**DKA**: Differentiated knowledge-acquisition
**DO**: Dandelion optimizer
**DOF**: Degree of freedom
**RL**: Random load
**k, n**: Region indices
**i**: Solution index
**x**: Area number
**T**_**st**_: Settling time
**MSL**: Multi-stage load
**OM**: Output measured
**LFC**: Load frequency control
**TD**: Time delay
**PV**: Photovoltaic energy unit
**RE**: Reflective evaluation
**RES**: Renewable energy source
**RUN**: Runge kutta optimizer
**AVR**: Automatic voltage regulator
**t**: Iteration counter
**POS**: Peak overshoot
**PUS**: Peak undershoot
